# Health Care Use and Expenditures Associated With Cardiac Rehabilitation Among Eligible Medicare Fee‐for‐Service Beneficiaries

**DOI:** 10.1161/JAHA.124.037811

**Published:** 2025-02-24

**Authors:** Lisa M. Pollack, Anping Chang, Jun Soo Lee, Thomas Shaffer, Hilary K. Wall, Clinton A. Brawner, Michael P. Thompson, Steven J. Keteyian, Devraj Sukul, Feijun Luo, Sandra L. Jackson

**Affiliations:** ^1^ Division for Heart Disease and Stroke Prevention, National Center for Chronic Disease Prevention and Health Promotion Centers for Disease Control and Prevention Atlanta GA USA; ^2^ Center for Medicare & Medicaid Innovation, Centers for Medicare & Medicaid Services Baltimore MD USA; ^3^ Division of Cardiovascular Medicine Henry Ford Health Detroit MI USA; ^4^ Department of Cardiac Surgery Michigan Medicine Ann Arbor MI USA; ^5^ Center for Healthcare Outcomes and Policy University of Michigan Ann Arbor MI USA; ^6^ Division of Cardiovascular Medicine, Department of Internal Medicine Michigan Medicine University of Michigan Health Ann Arbor MI USA

**Keywords:** cardiac rehabilitation, emergency department visits, expenditures, inpatient hospitalizations, Cardiovascular Disease

## Abstract

**Background:**

Cardiac rehabilitation (CR) can improve cardiovascular health. We identified whether CR participation was associated with fewer subsequent inpatient hospitalizations and emergency department visits and less Medicare and out‐of‐pocket expenditures, and whether outcomes varied by amount of participation.

**Methods:**

This retrospective study used Medicare fee‐for‐service claims data, including beneficiaries with a CR‐qualifying event in 2016. Participants attended ≥2 sessions of CR within 365 days of the event. Propensity score matching was used to identify CR‐eligible nonparticipants. Difference‐in‐differences analyses were used to compare differences in outcomes before (2014–2015) and after (2018–2019; 2‐year CR period=2016–2017) the CR period between participants and nonparticipants.

**Results:**

We identified 57 668 CR‐eligible beneficiaries after matching, with equal numbers of participants and nonparticipants. Nearly 65% of beneficiaries had a percutaneous coronary intervention, 33.5% had an acute myocardial infarction, 17.5% had a coronary artery bypass graft, and 16.8% had a heart valve repair/replacement. Compared with nonparticipants, participants had 47.6 fewer subsequent annual inpatient hospitalizations per 1000 beneficiaries (95% CI, −58.8 to −36.3) and $1005 lower subsequent annual Medicare expenditures per beneficiary (95% CI, −$1352 to −$659). Compared with no participation, medium participation (12–23 sessions), high participation (24–35 sessions), and CR completion (≥36 sessions) were associated with fewer inpatient hospitalizations and lower Medicare expenditures per year.

**Conclusions:**

CR was associated with fewer subsequent annual inpatient hospitalizations and lower subsequent annual Medicare expenditures. A higher amount of participation was associated with a further reduction in hospitalizations and expenditures. These findings can inform programs and policies that encourage CR participation.

**Nonstandard Abbreviations and Acronyms**
CCW VRDC: Chronic Conditions Data Warehouse Virtual Research Data Center
CMS: Centers for Medicare and Medicaid Services
CR: cardiac rehabilitation
DID: difference‐in‐differences
HCPCS: Healthcare Common Procedure Coding System

**Research Perspective**

**What Is New?**

- In this large, longitudinal cohort of Medicare fee‐for‐service beneficiaries, we found that cardiac rehabilitation (CR) participants, compared with nonparticipants, had, on average, 48 fewer subsequent inpatient hospitalizations per 1000 beneficiaries per year and $1005 lower subsequent Medicare expenditures per beneficiary per year.
- The observed reduction in expenditures could project to Medicare savings of $28.9 million if all CR‐eligible beneficiaries participated; overall, these findings can inform programs and policies that encourage CR participation.

**What Question Should Be Addressed Next?**

- What is the cost‐effectiveness of in‐person CR compared with virtual or hybrid CR programs?

Million Hearts is a national initiative co‐led by the Centers for Disease Control and Prevention and the Centers for Medicare and Medicaid Services (CMS).^1^ The goal of the initiative is to prevent 1 million cardiovascular events by 2026.^1^ One way to support this goal is by improving participation rates for cardiac rehabilitation (CR), a secondary prevention treatment that improves cardiovascular health following certain cardiac events or procedures.^1^

CR services are covered by CMS for beneficiaries following acute myocardial infarction (AMI), coronary artery bypass graft (CABG), current stable angina pectoris, heart valve repair or replacement, percutaneous coronary intervention (PCI; includes percutaneous transluminal coronary angioplasty or coronary stenting), heart or heart lung transplant, and stable chronic heart failure with reduced ejection fraction.^2^ CR participation is associated with a reduction in some medical resource utilization, including reductions in the risk of hospitalizations^3^, ^4^, ^5^ and hospital readmissions, although some findings have been mixed.^5^, ^6^ Participation in CR is also associated with health benefits, including reductions in cardiovascular mortality^3^ and all‐cause mortality^4^, ^7^, ^8^ and improvements in exercise capacity and quality of life.^3^ Previous studies of health care utilization are limited by older data^3^, ^4^, ^6^ or a follow‐up period of <1 year.^4^ In addition, less is known about the association between CR participation and subsequent health care expenditures. In a 2016 review and meta‐analysis, Anderson et al^3^ found that only 7 studies reported data on expenditures, with mixed findings, including no difference in total health care expenditures,^9^, ^10^, ^11^ lower health care expenditures,^12^ or higher health care expenditures^13^ among CR participants compared with nonparticipants. In addition to inconclusive findings, existing studies are limited by small sample size,^9^, ^11^, ^12^, ^13^, ^14^ study samples of only males,^12^ estimates based on data >15 years old,^9^, ^10^, ^11^, ^12^, ^13^, ^15^ and/or data from non–US hospital systems.^9^, ^11^, ^12^, ^13^, ^14^, ^16^ Furthermore, many existing studies include only a single cardiac condition, with AMI being the most common,^10^, ^13^, ^14^ which limits the ability to draw conclusions about whether CR is associated with less health care expenditures across the range of other CR‐qualifying conditions and procedures.^2^, ^17^

To address these gaps in the literature, the objectives of this study were to identify whether CR participation was associated with fewer subsequent annual inpatient hospitalizations and emergency department visits and reduced subsequent annual Medicare and out‐of‐pocket expenditures and whether these outcomes varied by amount of CR participation.

## METHODS

### Data

This retrospective cohort study used Medicare claims data (Parts A, B, and D), from the Chronic Conditions Data Warehouse Virtual Research Data Center (CCW VRDC), during 2014 to 2020. These data included beneficiary‐level medical utilization, expenditures, and enrollment from inpatient, outpatient, skilled nursing facility, home health, hospice, durable medical equipment, and prescription medications. Beneficiaries were followed longitudinally using a unique CCW beneficiary identifier. This research was considered exempt from institutional review board review under 45 Code of Federal Regulations 46.101[b] [5], which covers Department of Health and Human Services research and demonstration projects, which are designed to study, evaluate, or examine public benefit or service programs. The data that support the findings of this study cannot be shared due to data use agreements; researchers can reach out to CMS for data access. The methods used to generate the analytic cohort can be found on the Million Hearts webpage.^18^ This study follows the STROBE (Strengthening the Reporting of Observational Studies in Epidemiology) cohort reporting guidelines.^19^

### Analytic Cohort

#### Inclusion Criteria

The analytic cohort included US Medicare fee‐for‐service beneficiaries who had a CR‐qualifying event in 2016 (ie, AMI or a CABG surgery, heart valve repair or replacement [either surgical or transcatheter‐based therapy], PCI [includes percutaneous transluminal coronary angioplasty or coronary stenting], or heart or heart‐lung transplant). The CR‐qualifying events were identified using *International Classification of Diseases, Ninth Revision* (*ICD‐9*) or *International Classification of Diseases, Tenth Revision, Clinical Modification* (*ICD‐10‐CM*) diagnosis (first‐ or second‐listed code) or procedural codes (any location) on inpatient claims, or a current procedural terminology code (any location) on outpatient or provider claims (Table S1).^18^, ^20^ We did not include beneficiaries with current stable angina pectoris or stable chronic heart failure with reduced ejection fraction because of differences in how the conditions are identified in claims data^18^ and medical and device therapies and overall care paths for those two conditions. Beneficiaries had to be 67 years and older as of January 1, 2016 (to allow for a 2‐year pre‐CR period, 2014–2015) and continuously enrolled in Medicare Parts A, B, and D from January 1, 2014, through 3 years after the CR period (2016–2017) and to have survived for the duration of the study period (2014–2019) (Figure S1).^18^, ^20^, ^21^

#### Exclusion Criteria

Beneficiaries were excluded if they: (1) experienced a CR‐qualifying event or participated in traditional or intensive CR within 2 years before the CR‐qualifying event (pre‐CR period); (2) had ≥90 consecutive days in an inpatient acute care hospital, other inpatient hospital, or postacute care setting (extended hospital stay) within the pre‐CR period, or initiated an extended hospital stay within 21 days after the initial CR‐qualifying event; (3) received hospice care in the pre‐CR period or initiated hospice care within 21 days after the initial CR‐qualifying event^18^, ^20^, ^21^; or (4) had end‐stage renal disease in the pre‐CR period or after the CR‐qualifying event.^18^

### Measures

#### CR‐Qualifying Events

The CR‐qualifying events were categorized into the following nonmutually exclusive groups: AMI (with any procedure, no procedure); CABG (with AMI, no AMI); PCI (with AMI, no AMI); heart valve repair or replacement (with AMI, no AMI); heart or heart‐lung or ventricular assist device; and combination event (with AMI, no AMI). Beneficiaries with ≥1 CR‐qualifying event within 21 days (including events that occurred on the same day) were considered to have a combination event, consistent with previous methodology for assessing CR.^20^, ^21^ The categories are not mutually exclusive and thus will not sum to 100% because, for example, a beneficiary with an AMI followed by a PCI within 21 days would be considered to have had an AMI with any procedure, a PCI with AMI, and a combination event. If an individual had a subsequent event (n=6177, 5.3%), only the initial CR‐qualifying event was analyzed.

#### Identifying In‐Person Outpatient CR Claims

The presence of in‐person outpatient CR sessions (standard or intensive) were identified by Healthcare Common Procedure Coding System (HCPCS) codes within 365 days of the qualifying event index date (Table S1).^20^ For standard CR, Medicare covers up to two 1‐hour sessions per day, up to 36 sessions, over a period of up to 36 weeks (or 252 days).^2^ For intensive CR, Medicare covers up to six 1‐hour sessions per day, up to 72 sessions, during a period of up to 18 weeks (or 126 days).^22^ For single CR‐qualifying events, the index date was the inpatient hospital discharge date associated with the event or outpatient procedure date. For combination events, the inpatient hospital discharge date associated with the last qualifying event or the outpatient procedure date was used.^18^ Individual CR sessions on different dates were used to count the number of CR sessions. If >1 CR session occurred on the same day (9%), they were counted as a single session.^18^

#### CR Participants and Nonparticipants

We classified CR participants as those who initiated CR, defined as beneficiaries with a CR‐qualifying event who attended ≥2 sessions of outpatient CR within 365 days of the qualifying event index date.^18^ Nonparticipants were defined as beneficiaries with a CR‐qualifying event in 2016 who did not enroll in outpatient CR or had only one CR session within 1 year of discharge from the CR‐qualifying event. One CR session was classified as no participation because the first CR session is usually an orientation versus exercise session.

#### Amount of CR Participation

The amount of CR participation was measured as the total number of CR sessions attended by the beneficiary within 365 days of the qualifying event index date and then categorized as follows: nonparticipants (0 or 1 session), low (2–11 sessions), medium (12–23 sessions), high (24–35 sessions), and completion (≥36 sessions).^20^, ^23^

#### Patient‐Level Inpatient Hospitalizations, Emergency Department Visits, and Expenditures

To create mutually exclusive categories, the number of inpatient hospitalizations was defined as direct hospitalizations and emergency department (ED) visits resulting in hospitalization, and the number of ED visits was defined as ED visits not ending in inpatient hospitalization.

Medicare expenditures were defined as the sum of services paid to the provider for all inpatient, outpatient, skilled‐nursing facility, home health, hospice, durable medical equipment, and prescription drug services. Out‐of‐pocket expenditures were defined as patient cost‐sharing expenditures, including deductibles, copayments, and coinsurance from the same claim sources. To examine the composition of Medicare expenditures, we evaluated Medicare expenditures by claim type (inpatient, outpatient, Parts B and D, skilled nursing facility, durable medical equipment, home health, and hospice claims). Expenditures were adjusted to 2019 US dollars using the personal consumption expenditure price index from the Bureau of Economic Analysis.^24^

Cardiovascular disease–related inpatient hospitalizations, ED visits, and expenditures were defined as claims with an *ICD‐9*/*ICD‐10‐CM* diagnosis code for cardiovascular disease as the first‐listed or primary diagnosis code (Table S1).^25^

### Patient Characteristics

Demographic characteristics included age at the CR‐qualifying event index date (≤70, 71–75, 76–80, 81–85, >85 years), sex (male, female), race and ethnicity (non‐Hispanic White, non‐Hispanic Black, Hispanic, non‐Hispanic Asian, other/unknown), and dual Medicare and Medicaid coverage status, determined by CCW VRDC monthly indicators for dual enrollment (no dual enrollment, partial, or full dual enrollment). Geography included urbanicity of residence (metropolitan statistical area [population ≥50 000], micropolitan statistical area [population 10 000–50 000], rural area [population <10 000]) and the 9 US Census Divisions (New England, Mid Atlantic, South Atlantic, East North Central, East South Central, West North Central, West South Central, Mountain, Pacific). Comorbidities were identified in the pre‐CR period using the chronic conditions algorithms^26^ and presented individually and as a weighted index using the Charlson Comorbidity Index.^27^ We included the hierarchical condition category risk score, which is used by CMS as part of a risk‐adjustment model to account for differences in expected health expenditures of individuals.^28^, ^29^ The CR‐qualifying event inpatient length of stay (LOS) was defined as the difference between the CR‐qualifying event's first and last service dates (zero days for those with an outpatient event or procedure). Time to CR initiation (among CR participants) was measured as the number of days from the qualifying event index date to the second CR session date.^18^

### Statistical Analysis

#### Matching Procedure

To create well‐balanced groups, nonparticipants were matched to CR participants exactly on sex, race and ethnicity, age categories in 2016, urbanicity of residence, CR‐qualifying event type, and CR‐qualifying event date (at the monthly level to ensure similar timeframes for evaluating outcomes between matched groups) using SAS Proc PSMATCH (GREEDY 1:1 method on logit propensity score with a caliber=0.25).^30^ Propensity scores were derived from multivariate logistic regression on all other observable risk factors, including exact matched variables, US Census Division, dual enrollment, comorbidity conditions, Charlson Comorbidity Index, hierarchical condition category risk score, and LOS. We used standardized differences (ie, the difference between means divided by the pooled SD of the 2 groups) to describe the magnitudes of differences between CR participants and nonparticipants.^31^ Standardized differences <10% indicated a negligible difference between the 2 groups.^31^, ^32^

#### Association Between CR and Inpatient Hospitalizations, ED Visits, and Expenditures

Difference‐in‐differences (DID) analyses were used to compare differences in inpatient hospitalizations, ED visits, and expenditures before (2014–2015) and after (2018–2019; 2‐year CR period=2016–2017) the CR period between CR participants and nonparticipants (reference group) (see Figure S2 for study timeline). DID analyses were also used to compare differences in inpatient hospitalizations, ED visits, and expenditures before and after the CR period between CR participants (by the 4 categories of amount of CR participation) and nonparticipants (reference group). Our DID model can be summarized as follows:

$$\begin{array}{c} Y_{i}=a+\beta \left(\mathrm{CR}\;{\text{indicator}}_{i}\right)+\gamma \left({\text{Time}}_{i}\right)\\ \;+\emptyset \left(\mathrm{CR}\;\text{indicator}\times \text{Time}\right)+\delta \left(X_{i}\right)+\varepsilon_{i} \end{array}$$

where, *Y*_i_=outcome variable; *i*=individuals; Time=indicator for time trend (=1 for post‐CR period, =0 for pre‐CR period); *X*_*i*_=individual‐level control variables; *ε*_*i*_=error; Ø=DID estimate (ie, interaction between the indicators for CR and time).

Results are presented for any CR‐qualifying event (ie, all CR‐qualifying events combined) and stratified by CR‐qualifying event type, including combination events. An individual who had a combination event (eg, AMI followed by a PCI) would have been included in the any CR‐qualifying event model, and in the models stratified by CR‐qualifying event type (ie, PCI, AMI, and combination in this example). Inpatient hospitalizations and ED visits are presented per 1000 beneficiaries per year and expenditures are presented per beneficiary per year. As a visual indication that CR participants and nonparticipants had parallel trends in outcomes, we examined whether trends in the pre‐CR period were parallel to check against major violations in an assumption for DID analyses.^33^, ^34^ In adjusted analyses, for any CR‐qualifying event, we controlled for age, sex, race and ethnicity, dual enrollment status, urbanicity, US Census Division, comorbidities (individually), CR‐qualifying event type, hierarchical condition category risk score, and CR‐qualifying event LOS. In stratified analyses, we controlled for all variables except for the CR‐qualifying events.

### Sensitivity Analysis

We conducted several sensitivity analyses. First, we examined the association between CR participation and total medical expenditures, defined as the sum of Medicare and out‐of‐pocket expenditures. We did not include payments made by a primary payer other than Medicare in our definition for total expenditures because 99.7% of the cohort had a zero value for primary payments. Second, to examine whether conclusions remained consistent with the main analyses after being unable to retain all CR participants during the matching procedure, we replicated all analyses for the unmatched cohort (N=117 211). Third, because women as a group have less enrollment and participation in CR than men,^35^ triple difference models were used to compare differences in outcomes by sex. To obtain the triple difference estimates, we used generalized linear models in SAS.^36^ Fourth, we compared differences in outcomes before and after the CR period including year 2020 (ie, including a 3‐year post‐CR period, 2018–2020), because, while the pandemic may have affected observed outcomes, it may not have affected CR participants and nonparticipants differentially. Finally, we conducted a sensitivity analysis excluding the top 1% of expenditures to confirm that the expenditure analyses were not overly sensitive to the values of the highest outcomes. All analyses were performed with SAS version 9.4 (SAS Institute Inc.) or STATA release 18 (StataCorp LLC.).

## RESULTS

### CR Participation Status and Overall Patient Characteristics

Among 117 211 Medicare fee‐for‐service beneficiaries aged ≥67 years with a CR‐qualifying event in 2016 (Figure S1), 35.0% were CR participants (n=41 065) and 65.0% were nonparticipants (n=76 146) before matching (Table S2). After matching, there were 57 668 Medicare fee‐for‐service beneficiaries, with equal numbers of CR participants and nonparticipants (n=28 834) (Table 1). Among the matched cohort, 50.0% of beneficiaries had no participation (0 or 1 CR session, n=28 834), 7.3% had low participation (2–11 CR sessions, n=4209), 10.2% had medium participation (12–23 CR sessions, n=5868), 17.1% had high participation (24–35 CR sessions, n=9837), and 15.5% completed CR (≥36 CR sessions, n=8920). For CR participants, the mean time to CR initiation was ≈47 days (SD, 51 days) (Table 1).

**Table 1:** Characteristics of Medicare FFS Beneficiaries With a CR‐Qualifying Event in 2016, Overall and by CR Participation Status, 2014–2019, CMS, CCW (Matched Cohort)

	Overall		CR nonparticipants 0 or 1 CR session		CR participants ≥2 CR sessions		Standard difference*
	No.	%	No.	%	No.	%	
Total	57 668	100.00	28 834	100.00	28 834	100.00	…
Age, y†							
≤70	14 868	25.78	7434	25.78	7434	25.78	0.0000
71–75	19 712	34.18	9856	34.18	9856	34.18	0.0000
76–80	13 612	23.60	6806	23.60	6806	23.60	0.0000
81–85	6938	12.03	3469	12.03	3469	12.03	0.0000
>85	2538	4.40	1269	4.40	1269	4.40	0.0000
Sex†							
Male	35 712	61.93	17 856	61.93	17 856	61.93	0.0000
Female	21 956	38.07	10 978	38.07	10 978	38.07	0.0000
Race and ethnicity‡							
Non‐Hispanic White	55 470	96.19	27 735	96.19	27 735	96.19	0.0000
Non‐Hispanic Black	736	1.28	368	1.28	368	1.28	0.0000
Hispanic	634	1.10	317	1.10	317	1.10	0.0000
Non‐Hispanic Asian and Pacific Islander	364	0.63	182	0.63	182	0.63	0.0000
Other/unknown	464	0.80	232	0.80	232	0.80	0.0000
Dual Medicare and Medicaid coverage‡ ^,^ §							
No dual enrollment	56 192	97.44	28 036	97.23	28 156	97.65	−0.0264
Partial/full dual enrollment	1476	2.56	798	2.77	678	2.35	0.0264
Urbanicity of residence‡							
Metropolitan statistical area, population ≥50 000	45 342	78.63	22 671	78.63	22 671	78.63	0.0000
Micropolitan statistical area, population 10 000–50 000	7104	12.32	3552	12.32	3552	12.32	0.0000
Rural area, population <10 000	5222	9.06	2611	9.06	2611	9.06	0.0000
US Census Division							
New England	3011	5.22	1442	5.00	1569	5.44	0.0198
Mid Atlantic	7420	12.87	4004	13.89	3416	11.85	−0.0609
South Atlantic	11 018	19.11	5234	18.15	5784	20.06	0.0485
East North Central	5077	8.80	2347	8.14	2730	9.47	0.0469
East South Central	12 792	22.18	6434	22.31	6358	22.05	−0.0063
West North Central	3874	6.72	2014	6.98	1860	6.45	−0.0213
West South Central	5772	10.01	2989	10.37	2783	9.65	−0.0238
Mountain	3526	6.11	1677	5.82	1849	6.41	0.0249
Pacific	5178	8.98	2693	9.34	2485	8.62	−0.0252
Comorbidity conditions‡							
Alzheimer disease	623	1.08	323	1.12	300	1.04	−0.0077
Anemia	29 016	50.32	14 736	51.11	14 280	49.52	−0.0316
Asthma	7853	13.62	3972	13.78	3881	13.46	−0.0092
Atrial fibrillation and flutter	9559	16.58	4847	16.81	4712	16.34	−0.0126
Benign prostatic hyperplasia	17 312	30.02	8670	30.07	8642	29.97	−0.0021
Cancer‖	9247	16.03	4601	15.96	4646	16.11	0.0043
Cataract	41 505	71.97	20 540	71.24	20 965	72.71	0.0328
Chronic kidney disease	15 862	27.51	8148	28.26	7714	26.75	−0.0337
Chronic obstructive pulmonary disease	13 481	23.38	6958	24.13	6523	22.62	−0.0357
Depression, bipolar, or other mood disorders	14 934	25.90	7569	26.25	7365	25.54	−0.0162
Diabetes	25 086	43.50	12 742	44.19	12 344	42.81	−0.0278
Glaucoma	13 353	23.15	6584	22.83	6769	23.48	0.0152
Heart failure and nonischemic heart disease	14 311	24.82	7432	25.78	6879	23.86	−0.0444
Hip/pelvic fracture	863	1.50	437	1.52	426	1.48	−0.0031
Hyperlipidemia	51 680	89.62	25 879	89.75	25 801	89.48	−0.0089
Hypertension	51 163	88.72	25 703	89.14	25 460	88.30	−0.0266
Hypothyroidism	15 070	26.13	7535	26.13	7535	26.13	0.0000
Ischemic heart disease	37 111	64.35	19 025	65.98	18 086	62.72	−0.0680
Osteoporosis with or without pathological fracture	8249	14.30	4078	14.14	4171	14.47	0.0092
Rheumatoid arthritis/osteoarthritis	35 270	61.16	17 577	60.96	17 693	61.36	0.0083
Stroke/transient ischemic attack	7762	13.46	3978	13.80	3784	13.12	−0.0197
Charlson Comorbidity Index							
0	46 861	81.26	23 270	80.70	23 591	81.82	0.0285
1	3853	6.68	1921	6.66	1932	6.70	0.0015
2	2724	4.72	1416	4.91	1308	4.54	−0.0177
3	1798	3.12	945	3.28	853	2.96	−0.0184
4	2432	4.22	1282	4.45	1150	3.99	−0.0228
CR‐qualifying event†							
PCI#	37 200	64.51	18 600	64.51	18 600	64.51	0.0000
PCI, without AMI (no other procedures)	22 806	39.55	11 403	39.55	11 403	39.55	0.0000
PCI, without AMI (could have other procedures)	148	0.26	74	0.26	74	0.26	0.0000
PCI, with AMI (could have other procedures)	14 246	24.70	7123	24.70	7123	24.70	0.0000
AMI¶	19 316	33.50	9658	33.50	9658	33.50	0.0000
AMI (without procedure)	2888	5.01	1444	5.01	1444	5.01	0.0000
AMI (with procedure)	16 428	28.49	8214	28.49	8214	28.49	0.0000
CABG¶	10 108	17.53	5054	17.53	5054	17.53	0.0000
CABG, without AMI (no other procedures)	5912	10.25	2956	10.25	2956	10.25	0.0000
CABG, without AMI (could have other procedures)	1896	3.29	948	3.29	948	3.29	0.0000
CABG, with AMI (could have other procedures)	2300	3.99	1150	3.99	1150	3.99	0.0000
Heart valve repair or replacement¶	9708	16.83	4854	16.83	4854	16.83	0.0000
Heart valve repair, without AMI (no other procedures)	7632	13.23	3816	13.23	3816	13.23	0.0000
Heart valve repair, without AMI (could have other procedures)	1956	3.39	978	3.39	978	3.39	0.0000
Heart valve repair, with AMI (could have other procedures)	120	0.21	60	0.21	60	0.21	0.0000
Heart or heart‐lung transplant or VAD#	#	#	#	#	#	#	NA
Combination event¶ ^,^ **	18 428	31.96	9214	31.96	9214	31.96	0.0000
Combination event, without AMI	2000	3.47	1000	3.47	1000	3.47	0.0000
Combination event, with AMI	16 428	28.49	8214	28.49	8214	28.49	0.0000

	Overall		CR nonparticipants 0 or1 CR session		CR participants ≥2 CR sessions		Standardized difference*
	Mean	SD	Mean	SD	Mean	SD	
Age, y	74.81	5.56	74.84	5.60	74.79	5.52	−0.0101
HCC risk score‡	1.03	0.77	1.05	0.77	1.02	0.76	−0.0422
CR‐qualifying event LOS†	7.97	12.52	8.13	12.90	7.80	12.13	−0.0257
Time to CR initiation, d†	…	…	…	…	46.85	50.78	…

AMI indicates acute myocardial infarction; CABG, coronary artery bypass graft; CCW, Chronic Conditions Data Warehouse; CMS, Centers for Medicare and Medicaid Services; CR, cardiac rehabilitation; FFS, fee‐for‐service; HCC, hierarchical condition category; LOS, length of stay; NA, not applicable; PCI, percutaneous coronary intervention; and VAD, ventricular assist device.

* Standardized differences <10% indicated a negligible difference between the 2 groups.

^†^ Identified in the CR period (2016–2017).

^‡^ Identified in the pre‐CR period (2014–2015).

^§^ Dual eligible only includes patients aged ≥65 years, per inclusion criteria.

^‖^ Cancerous conditions include breast, colorectal, endometrial, lung, prostate, urologic (kidney, renal pelvis, and ureter).

^¶^ With or without AMI or other procedures.

^#^ Cell sizes <11 could not be reported.

** Beneficiaries with ≥1 CR‐qualifying event within 21 days, including events that occurred on the same day, were considered to have a combination event and treated separately.

The largest percentage of beneficiaries had a PCI (n=37 200, 64.5%), followed by an AMI (n=19 316, 33.5%), a CABG (n=10 108, 17.5%), and a heart valve repair or replacement (n=9708, 16.8%). We could not report the percentage of beneficiaries who had a heart or heart‐lung transplant or ventricular assist device because the numbers were too small. These results include those with combination events (n=18 428, 32.0%). Overall, the mean LOS was 8.0 days (SD, 12.9 days) for nonparticipants and 7.8 days (SD, 12.1 days) for CR participants (standardized difference <10%). Among those who had an outpatient event or procedure (n=16 540), the mean LOS was zero days as expected because outpatient events/procedures do not involve an overnight stay.

The mean age of CR participants and nonparticipants was ≈75 years after matching. By sex, 61.9% of beneficiaries were male and 38.1% were female. Most beneficiaries were non‐Hispanic White (96.2%), followed by non‐Hispanic Black (1.3%), Hispanic (1.1%), other/unknown race and ethnicity (0.8%), and non‐Hispanic Asian and Pacific Islander (0.6%). Most beneficiaries resided in a metropolitan area (78.6%), followed by micropolitan (12.3%) and rural areas (9.1%).

### Unadjusted Trends

Unadjusted trends in inpatient hospitalizations, ED visits, and expenditures by CR participation status are presented in Table 2. Among the matched cohort, pretreatment trends among CR participants compared with nonparticipants were parallel for number of ED visits. For inpatient hospitalizations and Medicare expenditures, pretreatment trends for nonparticipants were slightly upward, compared with those for CR participants, representing slightly increased inpatient care utilization among nonparticipants.

**Table 2:** Unadjusted Outcomes for Any CR‐Qualifying Event by CR Participation Status Relative to the 2016–2017 CR Period

	Pre‐CR Year 2 (2014)		Pre‐CR Year 1 (2015)		Post‐CR Year 1 (2018)		Post‐CR Year 2 (2019)		Difference in means between pre‐ and post‐CR*		
	Mean	SD	Mean	SD	Mean	SD	Mean	SD	Mean	LCL	UCL
Matched cohort											
Nonparticipants											
Matched Cohort IP hospitalizations, No.†	176	525	188	542	393	882	447	983	−238	−246	−229
ED visits, No.†	333	801	379	899	576	1172	613	1218	−239	−251	−227
Medicare expenditures, $‡	8190	13 738	9155	14 601	16 660	27 105	19 136	32 693	−8788	−9064	−8512
Out‐of‐pocket expenditures, $‡ ^,^ §	2210	2511	2384	3196	3426	4142	3668	4782	−1125	−1170	−1081
Total medical expenditures, $‡ ^,^ §	10 716	15 894	11 881	17 087	20 542	30 647	23 415	36 895	−10 100	−10 414	−9786
CR participants											
IP hospitalizations, No.†	170	509	176	518	338	789	388	875	−190	−198	−182
ED visits, No.†	316	740	366	804	559	1068	604	1176	−241	−252	−229
Medicare expenditures, $‡ ^,^ §	8167	13 067	9012	14 106	15 648	24 568	17 979	28 561	−7783	−8032	−7534
Out‐of‐pocket expenditures, $‡ ^,^ §	2279	2456	2452	2825	3476	4142	3725	5215	−1107	−1152	−1061
Total medical expenditures, $‡ ^,^ §	10 788	15 252	11 831	16 449	19 640	28 008	22 394	32 591	−9118	−9404	−8833
Unmatched cohort											
Nonparticipants											
IP hospitalizations, No.†	218	603	239	632	463	978	532	1084	−269	−275	−263
ED visits, No.†	421	1063	484	1151	692	1455	734	1479	−261	−270	−251
Medicare expenditures, $‡ ^,^ §	9584	15 645	10 608	16 253	18 940	28 342	21 896	33 183	−9809	−9987	−9631
OOP expenditures, $‡ ^,^ §	2315	2761	2494	3038	3591	4258	3863	4724	−1192	−1220	−1164
Total medical expenditures, $‡ ^,^ §	12 200	17 824	13 422	18 633	22 967	31 862	26 341	37 214	−11 182	−11 383	−10 981
CR participants											
IP hospitalizations, No.†	162	492	174	513	325	770	373	861	−181	−188	−175
ED visits, No.†	310	740	359	803	550	1078	596	1178	−238	−248	−229
Medicare expenditures, $‡ ^,^ §	7908	12 788	8885	14 104	15 042	23 876	17 317	27 825	−7350	−7554	−7146
OOP expenditures, $† ^,^ §	2207	2403	2408	2913	3364	3986	3620	4947	−1059	−1095	−1022
Total medical expenditures, $‡ ^,^ §	10 446	14 905	11 652	16 459	18 894	27 206	21 595	31 754	−8617	−8851	−8384

CR indicates cardiac rehabilitation; ED, emergency department; IP, inpatient; LCL, lower confidence limit; OOP, out‐of‐pocket; and UCL, upper confidence limit.

* *t* test for difference in means between pre‐ and post‐CR.

^†^ Number per 1000 beneficiaries.

^‡^ Expenditures per beneficiary.

^§^ Part D claims accounted for >32% of total medical expenditures in the pre‐CR period (32.3% for CR participants and 34.3% for nonparticipants) and ≈30% in the post‐CR period (29.8% for CR participants and 31.0% for nonparticipants).

### Association Between CR Participation and Subsequent Inpatient Hospitalizations, ED Visits, and Expenditures (Adjusted)

**Figure 1:**
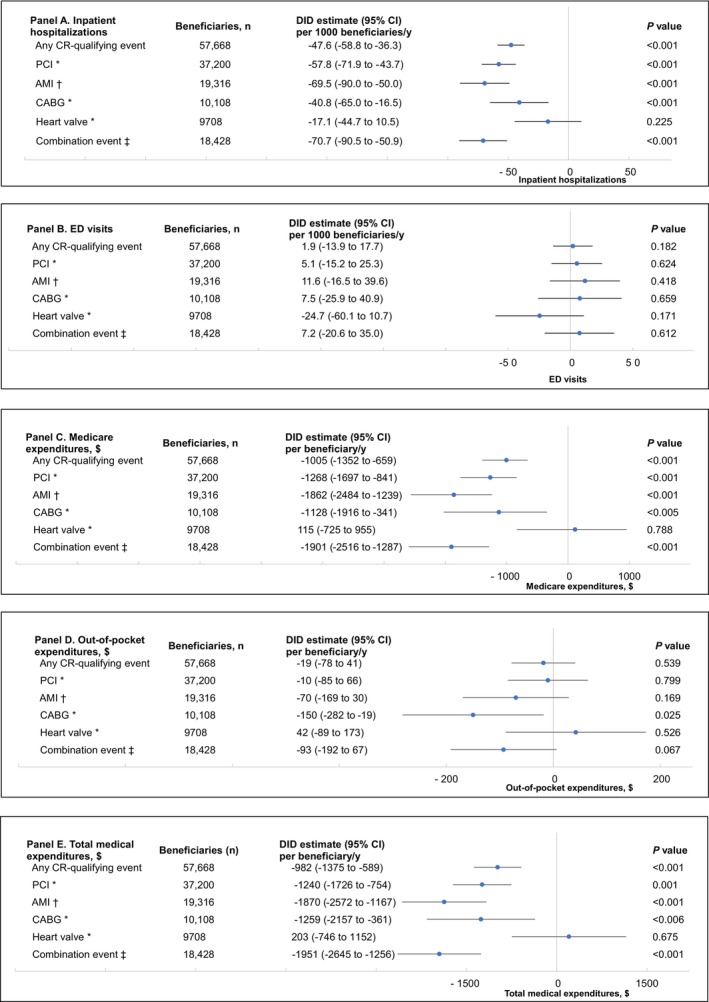
Association between CR participation and subsequent annual inpatient hospitalizations and ED visits (per 1000 beneficiaries) and expenditures (per beneficiary), for any CR‐qualifying event and stratified by event type (adjusted DID estimates, matched cohort). DID analyses were used to compare differences in inpatient hospitalizations (**A**), ED visits (**B**), Medicare expenditures (**C**), out‐of‐pocket expenditures (**D**), and total medical expenditures (**E**) before (2014–2015) and after (2018–2019; 2‐year CR period=2016–2017) the CR period between CR participants and nonparticipants (reference group). Models for any CR‐qualifying event controlled for age, sex, race and ethnicity, dual enrollment status, urbanicity, US Census Division, comorbidities, CR‐qualifying event (AMI [with or without procedure], CABG [with or without AMI], combination event [with or without AMI], heart valve repair or replacement [with or without AMI], PCI [with or without AMI]), HCC risk score, and primary qualifying event LOS. Models for PCI, AMI, CABG, heart valve repair or replacement, and combination procedure controlled for age, sex, race and ethnicity, dual enrollment status, urbanicity, US Census Division, comorbidities, HCC risk score, and primary qualifying event LOS. *PCI, CABG, and heart valve repair or replacement can be with or without AMI or other procedures. ^†^AMI can be with or without procedure. ^‡^Combination event can be with or without AMI. AMI indicates acute myocardial infarction; CABG, coronary artery bypass graft; CR, cardiac rehabilitation; DID, difference‐in‐difference; ED, emergency department; HCC, hierarchical condition category; LOS, length of stay; and PCI, percutaneous coronary intervention.

Figure 1 (and Tables S3 through S8) show the adjusted DID estimates for the association between CR and subsequent inpatient hospitalizations, ED visits, and expenditures. Among all eligible diagnoses, for every 1000 beneficiaries, CR participants had ≈47.6 fewer subsequent inpatient hospitalizations per year (−47.6 [95% CI, −58.8 to −36.3]) compared with nonparticipants (Figure 1A). Among all eligible diagnoses, for every beneficiary, CR participants had $1005 lower subsequent Medicare expenditures per year (−$1005 [95% CI, −$1352 to −$659]) compared with nonparticipants (Figure 1C).

When stratified by CR‐qualifying event type (Figure 1A through 1E), for those who had a combination event, compared with nonparticipants, CR participants had 71 fewer subsequent inpatient hospitalizations per 1000 beneficiaries per year (−70.7 [95% CI, −90.5 to −50.9]) and $1901 lower subsequent Medicare expenditures per beneficiary per year (−$1901 [95% CI, −$2516 to −$1287]). For those who had an AMI event, compared with nonparticipants, CR participants had ≈70 fewer subsequent inpatient hospitalizations per 1000 beneficiaries per year (−69.5 [95% CI, −90.0 to −50.0]) and $1862 lower subsequent Medicare expenditures per beneficiary per year (−$1862 [95% CI, −$2484 to −$1239]). For those who had a PCI, CR participants had ≈58 fewer subsequent inpatient hospitalizations per 1000 beneficiaries per year (−57.8 [95% CI, −71.9 to −43.7]) and $1268 lower subsequent Medicare expenditures per beneficiary per year (−$1268 [95% CI, −$1697 to −$841]). For those who had a CABG, CR participants had ≈41 fewer subsequent inpatient hospitalizations per 1000 beneficiaries per year (−40.8 [95% CI, −65.0 to −16.5]), $1128 lower subsequent Medicare expenditures per beneficiary per year (−$1128 [95% CI, −$1916 to −$341]), and $150 lower subsequent out‐of‐pocket expenditures per beneficiary per year (−$150 [95% CI, −$282 to −$19]). Findings for inpatient hospitalizations (Figure 1A) and Medicare expenditures (Figure 1C) for heart valve repair or replacement were not statistically significant. Findings for ED visits were not statistically significant (Figure 1B). Except for CABG, findings for out‐of‐pocket expenditures were not statistically significant (Figure 1D).

### Association Between Amount of CR Participation and Subsequent Inpatient Hospitalizations, ED Visits, and Expenditures (Adjusted)

**Figure 2:**
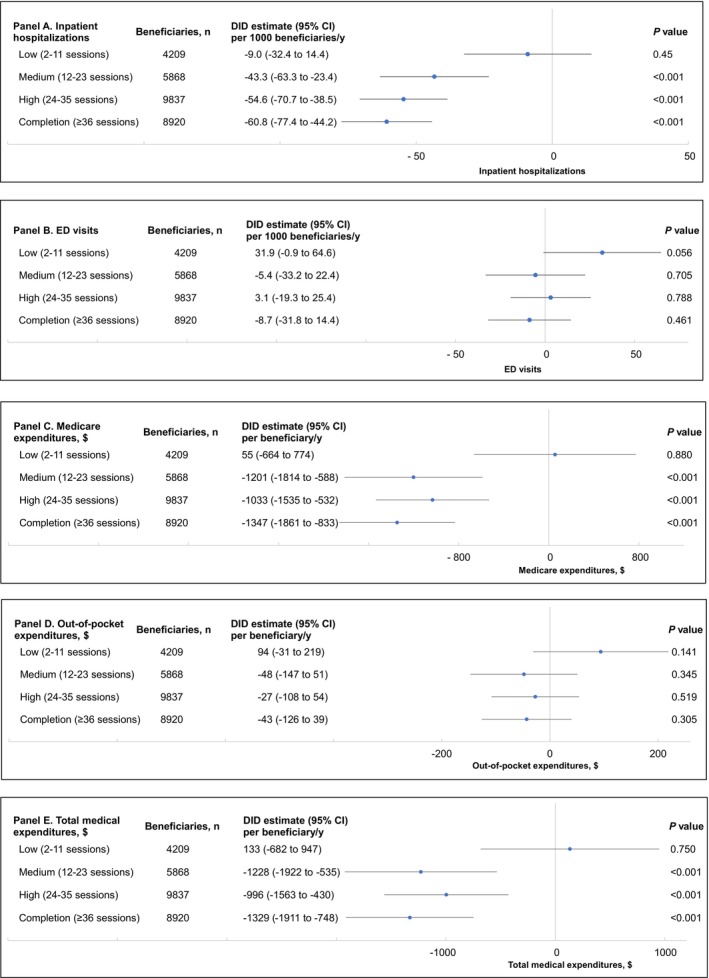
Association between amount of CR participation and subsequent annual inpatient hospitalizations and emergency department visits (per 1000 beneficiaries) and expenditures (per beneficiary), for any CR‐qualifying event (adjusted DID estimates, matched cohort). DID analyses were used to compare differences in inpatient hospitalizations (**A**), ED visits (**B**), Medicare expenditures (**C**), out‐of‐pocket expenditures (**D**), and total medical expenditures (**E**) before (2014–2015) and after (2018–2019; 2‐year CR period=2016–2017) the CR period between CR participants (by amount of CR participation: 2–11, 12–23, 24–35, ≥36 CR sessions) and nonparticipants (reference group [n=28 834, 50.0%]). All models controlled for age, sex, race and ethnicity, dual enrollment status, urbanicity, US Census Division, comorbidities, CR‐qualifying event (AMI [with or without procedure], CABG [with or without AMI], combination event [with or without AMI], heart valve repair or replacement [with or without AMI], PCI [with or without AMI]), hierarchical condition category risk score, and primary qualifying event LOS. AMI indicates acute myocardial infarction; CABG, coronary artery bypass graft; CR, cardiac rehabilitation; DID, difference‐in‐difference; ED, emergency department; LOS, length of stay; and PCI, percutaneous coronary intervention.

Figure 2 (and Tables S9 through S13) show the adjusted DID estimates for the association between amount of CR participation and subsequent inpatient hospitalizations, ED visits, and expenditures. A shows an inverse association between increasing amounts of CR participation and fewer subsequent inpatient hospitalizations per 1000 beneficiaries per year compared with nonparticipants (0 or 1 session): 12–23 sessions (medium participation: −43.3 [95% CI, −63.3 to −23.4]), 24–35 sessions (high participation: −54.6 [95% CI, −70.7 to −38.5]), or ≥36 CR sessions (completion: −60.8 [95% CI, −77.4 to −44.2]). The lowest participation category (2–11 sessions) was not statistically significant (−9.0 [95% CI, −32.4 to 14.4]).

Findings for ED visits (Figure 2B) and out‐of‐pocket expenditures (Figure 2D) by amount of CR participation were not statistically significant. Figure 2C shows that overall, compared with nonparticipants (0 or 1 session), CR participants had lower subsequent annual Medicare expenditures across the latter 3 levels of CR participation, from 12 to 23 sessions (medium participation: −$1201 [95% CI, −$1814 to −$588]), 24–35 sessions (high participation: −$1033 [95% CI, −$1535 to −$532]), to ≥36 CR sessions (completion: −$1347 [95% CI, −$1861 to −$833]).

### Association Between CR Participation and Medicare Expenditures by Claim Type for the Top Expenditures (Adjusted)

For both CR participants and nonparticipants, >90% of the Medicare expenditures were concentrated in 5 types of claims: inpatient, hospital outpatient, Part B, Part D, and skilled‐nursing facility claims. For any CR‐qualifying event, compared with nonparticipants, CR participants had $685 lower subsequent annual inpatient expenditures per beneficiary (−$685 [95% CI, −$881 to −$489]) and $260 lower subsequent annual skilled‐nursing facility expenditures per beneficiary (−$260 [95% CI, −$327 to −$194]), and $228 higher subsequent annual outpatient expenditures per beneficiary ($228 [95% CI, $93–$363]) (Table S14).

### Association Between CR Participation and Subsequent Cardiovascular Disease‐Related Inpatient Hospitalizations, ED Visits, and Expenditures (Adjusted)

For any CR‐qualifying event, compared with nonparticipants, CR participants had fewer subsequent cardiovascular disease–related inpatient hospitalizations per 1000 beneficiaries per year (−14.6 [95% CI, −20.4 to −9.0]) (Table S15). The CR participants had $273 lower subsequent total medical expenditures per beneficiary per year (−$273 [95% CI, −$427 to −$119]), compared with nonparticipants (Table S15). Findings for cardiovascular disease–related ED visits were not statistically significant (Table S15).

### Sensitivity Analysis

All findings for total medical expenditures were similar to our findings for Medicare expenditures (Figures 1E and 2E; Tables S3 through S8 and S13). All analyses for the unmatched cohort corroborated the main analyses, although, due to the imbalance between groups, the differences between groups were higher in the unmatched cohort (Tables S3 through S13 and S15). When comparing differences in outcomes by sex, findings were not statistically significant (Table S16). All findings with year 2020 included were consistent with the main findings (Table S17). Our findings for expenditures remained robust after excluding the top 1% of expenditures (Table S18).

## DISCUSSION

In this large, longitudinal cohort of Medicare fee‐for‐service beneficiaries, we found that CR participants, compared with nonparticipants, had, on average, 48 fewer subsequent inpatient hospitalizations per 1000 beneficiaries per year and $1005 lower subsequent Medicare expenditures per beneficiary per year. When stratified by CR‐qualifying event type, CR participation was associated with: (1) a reduction in number of inpatient hospitalizations, ranging from a reduction of 41 to 71 per 1000 beneficiaries per year, and (2) lower Medicare expenditures, ranging from $1128 to $1901 lower expenditures per beneficiary per year.

A higher amount of CR participation was associated with fewer subsequent inpatient hospitalizations after a threshold of 12 was attained. In addition, a higher amount of CR participation was associated with lower subsequent Medicare expenditures up to 24 to 35 CR sessions, after which effects leveled off. Further, for any CR‐qualifying event, compared with nonparticipants, CR participants experienced 15 fewer subsequent cardiovascular disease–related inpatient hospitalizations per 1000 beneficiaries per year. On the other hand, similar to Martin et al,^5^ CR participation was not associated with a reduction in ED visits. While we controlled for many confounding factors that may be associated with ED visits, including the CR‐qualifying event, LOS for the CR‐qualifying event (to capture information about the complexity/severity of the condition), underlying comorbidities, amount of CR participation (because low participation can limit the effectiveness of CR), we could not control for all associated factors, such as variability in program quality, access to quality CR programs, and psychosocial factors, potentially leading to unmeasured confounding.

Our results demonstrate that CR at any participation level was associated with fewer subsequent annual inpatient hospitalizations. These findings are consistent with older and smaller studies, which have shown benefits of CR participation.^4^, ^5^, ^6^, ^37^ For example, CR enrollment has been associated with fewer hospitalizations within 1 year of discharge.^4^ A systematic review published in 2021 that included 85 trials demonstrated that CR was associated with a reduction in all‐cause hospitalization up to 12 months of follow‐up.^37^ While we did not examine hospital readmission outcomes in this study, others have found that CR participation is associated with a reduction in all‐cause readmission, cardiovascular readmission, and noncardiovascular readmission.^6^

Moreover, a higher amount of CR participation was associated with additional subsequent reductions in annual inpatient hospitalizations. Our findings are consistent with other studies indicating that the benefits of CR are dose‐related.^38^ Authors of one study found that CR completion (ie, patients who completed their baseline assessment and 12‐week postrehabilitation assessment) versus noncompletion (ie, patients who completed their baseline assessment but did not return for their 12‐week assessment) was associated with a decreased risk of all‐cause hospitalization and cardiac hospitalization but not with ED visits.^5^ Others found that every additional CR session completed was associated with a 1.8% lower incidence rate of 1‐year cardiac readmission and a 2.0% lower incidence rate of all‐cause hospital readmission, controlling for age, sex, race, and number of CR sessions.^39^ The benefits of a higher amount of CR participation extend beyond health care utilization: studies have shown a relationship between a higher amount of CR participation and lower risk of death.^5^, ^7^, ^40^, ^41^ While our analysis demonstrates a lower percentage of CR completion (15.5%) compared with Keteyian et al.^38^ (2017) (27.6%), this difference can be attributed in part to the fact that we restricted our analytic cohort to individuals continuously enrolled in Medicare Parts A, B, and D for the duration of our study period (6 years; 2014 to 2019) and matched individuals on key characteristics. The previous analysis only restricted the analysis to Medicare Parts A and B for 12 months (vs 6 years) and did require matching techniques.

Our results demonstrate that CR at any participation level was associated with lower subsequent annual Medicare expenditures, and a higher amount of CR participation was associated with a reduction in annual Medicare expenditures. In line with our study, others found that compared with individuals who were not referred to CR, health care expenditures followed a dose–response relationship and were lowest in patients who had the highest CR participation levels (ie, attending ≥67% prescheduled classes).^40^ Several studies have found that CR is a cost‐effective intervention.^9^, ^11^, ^12^ Our findings differ from studies that found no difference in total health care expenditures^9^, ^10^, ^11^ or higher health care expenditures^13^ among CR participants compared with nonparticipants (or control or usual care groups). This might be attributable to differences in data used, sample size, study years, study design, or insurance policies and coverage, among other factors. Participation in CR was associated with $1005 lower subsequent Medicare expenditures per beneficiary per year. For the 28 834 beneficiaries in our study who had a CR‐qualifying event in 2016 and subsequently participated in CR, this may have yielded a savings of ≈$28.9 million per year for Medicare, although our study did not include expenditures to participate in CR. While 90% of expenditures in our study resulted from inpatient, hospital outpatient, Part B, Part D, and skilled‐nursing facility claims, lower expenditures among CR participants compared with nonparticipants were driven by lower annual inpatient and skilled‐nursing facility expenditures, 2 of the most acute forms of health care utilization. These expenditures are also related because Medicare generally only reimburses skilled‐nursing facility stays if they are proceeded by a qualifying 3‐day hospital admission. This study focused on eligible Medicare fee‐for‐service beneficiaries, so the results may not be generalizable to younger populations. However, our methods are reproducible using other administrative, claims‐based data sets.

Although 38.5% of eligible beneficiaries were removed from the study cohort due to our Medicare Part D continuous enrollment criteria for the duration of the study period (6 years), we did this because prescription drug expenditures are an important consideration among individuals with heart disease. In our study, at baseline (year 2016), nearly all individuals had medication expenditures (99.9%), ranging from $>0 to $611 329, and Part D claims accounted for ≈20% of total medical expenditures overall. If we had kept individuals in our study without continuous Medicare Part D enrollment, a large proportion of the expenditure calculation would have been missed—a main contribution of our study. As CR treatment includes medication education, pharmacist care/support, and access to free medication programs, one systematic review protocol describes examining the potential of CR programs to improve medication adherence in patients with cardiovascular disease.^42^

Not only is CR associated with many health benefits, including reductions in mortality (overall and cardiovascular‐related) and improvements in exercise capacity and quality of life,^3^, ^8^ our study shows an association with reductions in inpatient hospitalizations and expenditures. These reductions in expenditures will be relevant to Medicare and private insurers. However, CR is currently underutilized at the population level; Keteyian et al. found that only 28.6% of eligible patients initiated CR,^20^ and we found that only 35.0% of eligible patients participated in CR. CR enrollment and participation are lower than national goals owing to individual‐level, system‐level, and geographic factors.^43^ The Million Hearts CR Collaborative has committed to increasing CR participation to 70%.^38^, ^44^ Hospital administrators and CR champions can use strategies from the Million Hearts CR Change Package^45^ to help achieve the national goal for CR participation.^44^ Findings from this study could be used by CR leaders to empower health systems and administrators to encourage more CR participation (and sustained participation over 12 sessions).^44^ In addition, the cost estimates generated from our study could be used to examine the cost‐effectiveness of in‐person CR compared with virtual or hybrid CR programs, which increased in prevalence in response to the recent COVID pandemic.^46^

This is the first study, to our knowledge, to create a large, longitudinal cohort of individuals 67 years and older eligible for CR, diverse in sociodemographic and clinical characteristics, and to examine subsequent annual expenditures. We identified a wide range of patient, geographic, and clinical characteristics to create well‐balanced groups of CR and non‐CR participants and controlled for clinically relevant risk factors for all CR‐qualifying events.

Our study is not without limitations. First, because CR lowers mortality risk^7^, ^8^ and health care utilization and expenditures,^37^ we evaluated health care utilization and expenditures associated with CR among survivors, potentially underestimating the true effect of CR. A requirement of our using a DID approach meant maintaining balance over all observations and to ensure that we used a matched cohort among survivors. We performed a sensitivity analysis and confirmed that our matching procedure minimized any inherent differences between CR participants and nonparticipants and mortality rates between the 2 groups (27.5% in the unmatched cohort among survivors and decedents and 15.6% in the matched cohort among survivors), so that the matched cohort among survivors was not inherently different than the larger cohort including survivors and decedents.

Second, unmeasured confounding could have influenced our findings since we could not adjust for all health‐related decisions related to CR participation, but we excluded beneficiaries who may be too frail to participate in CR (ie, extended hospital stay, enrolled in hospice, or had end‐stage renal disease per exclusion criteria) and used propensity score matching to create balance groups and DID analyses to ameliorate any endogeneity issues. Third, while a 2‐year CR period could have underestimated the effect of CR for those who completed CR in the first year (as any treatment effect is usually strongest in the first year after the last CR session), we selected that timeframe to maintain the greatest number of beneficiaries in the analysis (60.5% of CR participants had their last CR session in 2016 and 39.5% in 2017). Fourth, our cohort comprised primarily White individuals, limiting the generalizability of our findings to populations of other races and ethnicities. Our data are consistent with others that have observed lower proportions of non‐White beneficiaries in Medicare data,^47^, ^48^ and in Medicare fee‐for‐service data in particular (the focus of our study),^49^ and lower rates of referral to CR and lower enrollment and completion rates among non‐White racial and ethnic groups.^50^ Fifth, while we could not assess for differences in social determinants of health between non‐Hispanic White and other racial and ethnic groups due to Z‐codes being unavailable in our baseline year,^51^ nonparticipants were matched to CR participants exactly on race and ethnicity to minimize the potential for unmeasured confounding.

## CONCLUSIONS

Participation in CR, at any participation level, was associated with fewer subsequent annual inpatient hospitalizations and lower subsequent annual Medicare expenditures. In addition, there was a dose–response relationship between the amount of CR participation and reductions in annual inpatient hospitalizations and expenditures. This analysis informs the treatment of individuals who experience a CR‐qualifying event and their care teams and supports programs and policies that encourage CR participation.

## Sources of Funding

Devraj Sukul receives funding from the Blue Cross Blue Shield of Michigan Foundation for his work in quality improvement through the Blue Cross Blue Shield of Michigan Cardiovascular Consortium (BMC2). Michael P. Thompson receives grant funding from the Agency for Healthcare Research and Quality (K01HS027830, R01HS028397) and funding from the Blue Cross Blue Shield of Michigan Value Partnerships initiative. Clinton A. Brawner and Steven J. Keteyian receive research grant support from National Institute on Aging and National Health, Lung, & Blood Institute.

## Disclosures

None.

## Supporting information

Tables S1–S18
Figures S1–S2
