# Supplementary material for: Health Care Use and Expenditures Associated With Cardiac Rehabilitation Among Eligible Medicare Fee‐for‐Service Beneficiaries
Source: J Am Heart Assoc. 2025 Feb 24;14(5):e037811. doi: 10.1161/JAHA.124.037811 (PMC12132668; doi:10.1161/JAHA.124.037811)
Supplement: Supplementary file 1 — Tables S1–S18 Figures S1–S2 [file JAH3-14-e037811-s001.pdf]

Supplemental Material

**Table S1.** Diagnosis and procedural codes	
**Cardiac rehabilitation qualifying events[*]**	
**AMI[†]**	
ICD-9-CM DX	410.00, 410.01, 410.02, 410.10, 410.11, 410.12, 410.20, 410.21, 410.22, 410.30, 410.31, 410.32, 410.40, 410.41, 410.42, 410.50, 410.51, 410.52, 410.60, 410.61, 410.62, 410.70, 410.71, 410.72, 410.80, 410.81, 410.82, 410.90, 410.91, 410.92
ICD-10-CM DX	I21.0, I21.01, I21.02, I21.09, I21.1, I21.11, I21.19, I21.2, I21.21, I21.29, I21.3, I21.4, I21.9, I21.A1, I21.A9, I22.0, I22.1, I22.2, I22.8, I22.9
**CABG**	
ICD-9-CM PR	36.10, 36.11, 36.12, 36.13, 36.14, 36.15, 36.16, 36.17, 36.19, 36.2
ICD-10-PCS	0210[‡], 0211[‡], 0212[‡], 0213[‡],<br>02700[§], 02710[§], 02720[§], 02730[§], 02C00[§], 02C10[§], 02C20[§], 02C30[§]
CPT/HCPCS	33510, 33511, 33512, 33513, 33514, 33516, 33533, 33534, 33535, 33536, S2205, S2206, S2207, S2208, S2209
**Heart valve repair/replacement**	
ICD-9-CM PR	35.00, 35.01, 35.02, 35.04, 35.05, 35.06, 35.07, 35.08, 35.09, 35.10, 35.11, 35.12, 35.13, 35.14, 35.20, 35.21, 35.22, 35.23, 35.24, 35.25, 35.26, 35.27, 35.28, 35.33, 35.96, 35.97, 35.99
ICD-10-PCS[‡]	027F, 027G, 027H, 027J, 02CF, 02CG, 02CH, 02CJ, 02NF, 02NG, 02NH, 02NJ, 02QF, 02QG, 02QH, 02QJ, 02RF, 02RG, 02RH, 02RJ, 02TH, 02UF, 02UG, 02UH, 02UJ, 02VG
CPT	33361-33366; 33390-33391;33400-33401; 33403-33406; 33410-33418; 33420; 33422; 33425-33427; 33430; 33460; 33463-33465; 33468; 33470-33472; 33474-33478; 33863; 0345T; 0483T; 0544T; 0545T; 0569T
**PCI[‖]**	
ICD-9-CM PR	00.66, 36.03, 36.04, 36.06, 36.07, 36.09
ICD-10-PCS[‡]	02703[§], 02704[§], 02713[§], 02714[§], 02723[§], 02724[§], 02733[§], 02734[§], 02C03[§], 02C04[§], 02C13[§], 02C14[§], 02C23[§], 02C24[§], 02C33[§], 02C34[§], 3E07017, 3E070PZ, 3E07317, 3E073PZ
CPT	92920, 92924, 92928, 92933, 92937, 92941, 92943, 92975
**Heart or heart-lung transplant or VAD**	
ICD-9-CM PR	33.6, 37.51, 37.52, 37.53, 37.54
ICD-10-PCS	02YA0Z0, 02YA0Z1, 02YA0Z2, 02RK0JZ, 02RL0JZ, 02WA0JZ
CPT	33927, 33928, 33945, 0051T, 0052T, 0053T
**Cardiac rehabilitation claims[#]**	
**Standard CR**	93797, 93798

**Intensive CR**	G0422, G0423
**Cardiovascular disease**	
ICD-9-CM DX	390-434, 436-448
ICD-10-CM DX	I00-I78

Abbreviations: AMI, acute myocardial infarction; CABG, coronary artery bypass graft; CPT, Current Procedural Terminology; DX, diagnosis; HCPCS, Healthcare Common procedure Coding System; ICD-9/-10-CM, International Classification of Diseases, 9th/10th edition, Clinical Modification; PCI, percutaneous coronary intervention; PCS, procedure coding system; PR, procedural; VAD, ventricular assist device; QE, qualifying event

[*]ICD-9-CM codes are in effect until September 30, 2015, and ICD-10-CM codes are in effect starting on October 1, 2015. Effective dates for ICD-9-CM, ICD-10-CM, and CPT codes may vary by year.
[†]Coded first or second listed ICD-9/-10-CM diagnosis code
[‡]Includes all codes with these as the first four identifiers
[§]Includes all codes with these as the first five identifiers
[‖]PCI includes percutaneous transluminal coronary angioplasty [PTCA] or coronary stenting and same-day discharge after an outpatient PCI
[#]A CR session is defined has having one of the HCPCS codes when billed with place of service codes of 11 (services provided in a physician’s office), 19 (off campus-outpatient hospital), or 22 (on campus-outpatient hospital)

**Table S2.** Characteristics of Medicare FFS beneficiaries with a CR-qualifying event in 2016, overall and by CR participation status, 2014–2019, CMS, CCW (unmatched cohort)							
	**Overall**		**CR non-Participants 0–1 CR Session**		**CR Participants ≥2 CR sessions**		**Stddif**[*]
	**N**	**%**	**N**	**%**	N	**%**	
**Total**	117,211	100.00	76,146	100.00	41,065	100.00	--
**Age, year**[†]							
≤70	27,769	23.69	16,816	22.08	10,953	26.67	0.1070
71–75	35,598	30.37	21,806	28.64	13,792	33.59	0.1070
76–80	27,060	23.09	17,351	22.79	9,709	23.64	0.0203
81–85	16,794	14.33	11,915	15.65	4,879	11.88	-0.1095
>85	9,990	8.52	8,258	10.84	1,732	4.22	-0.2531
**Sex**[‡]							
Male	66,904	57.08	41,308	54.25	25,596	62.33	0.1645
Female	50,307	42.92	34,838	45.75	15,469	37.67	-0.1645
**Race and ethnicity**[‡]							
Non-Hispanic White	104,298	88.98	66,144	86.86	38,154	92.91	0.2016
Non-Hispanic Black	4,266	3.64	3,358	4.41	908	2.21	-0.1231
Hispanic	4,171	3.56	3,410	4.48	761	1.85	-0.1504
Non-Hispanic Asian and Pacific Islander	2,289	1.95	1,814	2.38	475	1.16	-0.0931
Other/Unknown	2,187	1.87	1,420	1.86	767	1.87	0.2016
**Dual Medicare and Medicaid coverage**[‡,§]							
No dual enrollment	107,490	91.71	67,586	88.76	39,904	97.17	-0.3336
Partial/Full dual enrollment	9,721	8.29	8,560	11.24	1,161	2.83	0.3336
**Urbanicity of residence**[‡]							
Metropolitan statistical area, population ≥50,000	87,393	74.56	56,681	74.44	30,712	74.79	0.0081
Micropolitan statistical area, population 10,000-50,000	16,172	13.80	10,342	13.58	5,830	14.20	0.0178
Rural area, population <10,000	13,646	11.64	9,123	11.98	4,523	11.01	-0.0303
**U.S. Census Division**							
New England	5,749	4.90	3,538	4.65	2,211	5.38	0.0338

Mid Atlantic	17,251	14.72	12,623	16.58	4,628	11.27	-0.1538
	**Overall**		**CR non-Participants 0–1 CR Session**		**CR Participants ≥2 CR sessions**		**Stddif[*]**
South Atlantic	19,436	16.58	10,583	13.90	8,853	21.56	0.2016
East North Central	9,889	8.44	4,509	5.92	5,380	13.10	0.2466
East South Central	24,819	21.17	16,564	21.75	8,255	20.10	-0.0406
West North Central	8,676	7.40	6,247	8.20	2,429	5.92	-0.0895
West South Central	12,701	10.84	9,141	12.00	3,560	8.67	-0.1097
Mountain	6,491	5.54	4,083	5.36	2,408	5.86	0.0218
Pacific	12,199	10.41	8,858	11.63	3,341	8.14	-0.1174
**Comorbidity conditions[‡]**							
Alzheimer’s Disease	2,412	2.06	2,019	2.65	393	0.96	-0.1276
Anemia	64,723	55.22	44,914	58.98	19,809	48.24	-0.2167
Asthma	17,158	14.64	11,830	15.54	5,328	12.97	-0.0733
Atrial Fibrillation and Flutter	21,250	18.13	14,718	19.33	6,532	15.91	-0.0899
Benign Prostatic Hyperplasia	33,410	28.50	21,208	27.85	12,202	29.71	0.0411
Cancer[‖]	19,111	16.30	12,479	16.39	6,632	16.15	-0.0065
Cataract	85,418	72.88	55,667	73.11	29,751	72.45	-0.0148
Chronic Kidney Disease	36,171	30.86	25,480	33.46	10,691	26.03	-0.1630
Chronic Obstructive Pulmonary Disease	32,291	27.55	23,506	30.87	8,785	21.39	-0.2170
Depression, Bipolar, or Other Mood Disorders	33,283	28.40	23,237	30.52	10,046	24.46	-0.1359
Diabetes	55,010	46.93	37,653	49.45	17,357	42.27	-0.1445
Glaucoma	29,004	24.75	19,345	25.41	9,659	23.52	-0.0438
Heart Failure and Non-Ischemic Heart Disease	35,870	30.60	26,600	34.93	9,270	22.57	-0.2756
Hip/Pelvic Fracture	2,448	2.09	1,885	2.48	563	1.37	-0.0805
Hyperlipidemia	105,694	90.17	69,149	90.81	36,545	88.99	-0.0604
Hypertension	106,038	90.47	70,145	92.12	35,893	87.41	-0.1560
Hypothyroidism	33,105	28.24	22,736	29.86	10,369	25.25	-0.1033
Ischemic Heart Disease	79,544	67.86	54,924	72.13	24,620	59.95	-0.2593
Osteoporosis With or Without Pathological Fracture	20,534	17.52	14,875	19.53	5,659	13.78	-0.1549
Rheumatoid Arthritis/Osteoarthritis	74,127	63.24	49,256	64.69	24,871	60.56	-0.0853

Stroke/Transient Ischemic Attack	18,537	15.82	13,313	17.48	5,224	12.72	-0.1333
	**Overall**		**CR non-Participants 0–1 CR Session**		**CR Participants ≥2 CR sessions**		**Stddif**[*]
**Charlson Comorbidity Index**							
0	91,349	77.94	57,546	75.57	33,803	82.32	0.1659
1	8,521	7.27	5,864	7.70	2,657	6.47	-0.0480
2	6,326	5.40	4,476	5.88	1,850	4.51	-0.0619
3	4,491	3.83	3,288	4.32	1,203	2.93	-0.0744
4	6,524	5.57	4,972	6.53	1,552	3.78	-0.1246
**CR qualifying event**[†]							
**PCI, any**[#]	65,682	56.04	44,006	57.79	21,676	52.78	-0.1008
PCI, without AMI (no other procedures)	42,387	36.16	30,155	39.60	12,232	29.79	-0.2073
PCI, without AMI (could have other procedures)	802	0.68	459	0.60	343	0.84	0.0275
PCI, with AMI (could have other procedures)	22,493	19.19	13,392	17.59	9,101	22.16	0.1148
**AMI, any**[#]	46,021	39.26	32,527	42.72	13,494	32.86	-0.2043
AMI (without procedure)	18,437	15.73	16,841	22.12	1,596	3.89	-0.5631
AMI (with procedure)	27,584	23.53	15,686	20.60	11,898	28.97	0.1949
**CABG, any**[#]	20,158	17.20	8,049	10.57	12,109	29.49	0.4864
CABG, without AMI (no other procedures)	10,453	8.92	4,036	5.30	6,417	15.63	0.3423
CABG, without AMI (could have other procedures)	4,212	3.59	1,620	2.13	2,592	6.31	0.2093
CABG, with AMI (could have other procedures)	5,493	4.69	2,393	3.14	3,100	7.55	0.1968
**Heart valve repair or replacement, any**[#]	18,852	16.08	9,723	12.77	9,129	22.23	0.2510
Heart valve repair, without AMI (no other procedures)	13,622	11.62	7,472	9.81	6,150	14.98	0.1572
Heart valve repair, without AMI (could have other procedures)	4,419	3.77	1,826	2.40	2,593	6.31	0.1928
Heart valve repair, with AMI (could have other procedures)	811	0.69	425	0.56	386	0.94	0.0443
**Heart or heart-lung transplant or VAD**[**]	**	**	**	**	**	**	NA
**Combination event**[#,**]	32,287	27.55	17,635	23.16	14,652	35.68	0.2774
Combination event, without AMI	4,703	4.01	1,949	2.56	2,754	6.71	0.1983
Combination event, with AMI	27,584	23.53	15,686	20.60	11,898	28.97	0.1949

	Overall		CR non-Participants 0–1 CR Session		CR Participants ≥2 CR sessions		Stddif[*]
	Mean	SD	Mean	SD	Mean	SD	
**Age**	75.78	6.27	76.37	6.57	74.69	5.50	-0.2773
**HCC risk score[‡]**	1.14	0.84	1.21	0.87	1.00	0.75	-0.2593
**CR qualifying event LOS[†]**	9.09	13.75	8.48	13.78	10.22	13.62	0.1266
**Time to CR initiation, days[†]**	--	--	--	--	45.14	48.66	--

Abbreviations: AMI, acute myocardial infarction; BiV, biventricular; Centers for Medicare and Medicaid Services (CMS); Chronic Conditions Data Warehouse (CCW); CABG, coronary artery bypass graft surgery; CR, cardiac rehabilitation; FFS, fee-for-service; Hierarchical condition category (HCC); LOS, length of stay; NA, not applicable; PCI, percutaneous coronary intervention; SD, standard deviation; Stddif, standardized difference; TAVR, transcatheter aortic valve replacement/repair; VAD, ventricular assist device

[*]Standardized differences <10% indicated a negligible difference between the two groups
[†]Identified in the CR period (2016–2017)
[‡]Identified in the pre-CR period (2014–2015)
[§]Dual eligible only includes those aged ≥65 years, per inclusion criteria
[‖]Cancerous conditions include breast, colorectal, endometrial, lung, prostate, urologic (kidney, renal pelvis, and ureter)
[#]With or without AMI or other procedures
[**]Cell sizes <11 could not be reported
[‡‡]Beneficiaries with ≥1 CR-qualifying event within 21 days, including events that occurred on the same day, were considered to have a combination event and treated separately.

**Table S3.** Association between CR participation and inpatient hospitalizations and emergency department visits (per 1,000 persons) and expenditures (per person), for any CR-qualifying event (adjusted DID estimates)*					
	**Inpatient Hospitalizations**	**Emergency Department Visits**	**Medicare Expenditures, $**	**Out-of-pocket Expenditures, $**	**Total Medical Expenditures†, $**
Matched cohort‡					
DID estimate	-47.6***	1.9	-1,005***	-18.50	-982***
Confidence interval	(-58.8 - -36.3)	(-13.9 - 17.7)	(-1,352 - -659)	(-78 - 41)	(-1,375 - -589)
p-value	<0.001	0.812	<0.001	0.539	<0.001
Beneficiaries	57,668	57,668	57,668	57,668	57,668
R-squared	0.121	0.082	0.160	0.149	0.171
Mean control (pre-CR)	-317	-338.3	-6,374	-815	-7,244
Mean treated (pre-CR)	-312.4	-340.9	-5,973	-676	-6,667
Difference (pre-CR)	4.6	-2.6	401	140	577
Mean control (post-CR)	-79.5	-99.7	2,414	310	2,856
Mean treated (post-CR)	-122.5	-100.3	1,809	431	2,452
Difference (post-CR)	-43.0	-0.7	-604	121	-404
Unmatched cohort‡					
DID estimate	-87.7***	-22.1**	-2,459***	-133***	-2,564***
Confidence interval	(-96.8 - -78.7)	(-36.0 - -8.3)	(-2,722 - -2,195)	(-176 - -90)	(-2,860 - -2,267)
p-value	<0.001	0.002	<0.001	<0.001	<0.001
Beneficiaries	117,211	117,211	117,211	117,211	117,211
R-squared	0.130	0.084	0.180	0.155	0.188
Mean control (pre-CR)	-308.5	-361.4	-6,548	-644	-7,217
Mean treated (pre-CR)	-284.5	-358.3	-5,310	-437	-5,735
Difference (pre-CR)	24.0	3.2	1,237	207	1,482
Mean control (post-CR)	-40.0	-100.8	3,261	548	3,964
Mean treated (post-CR)	-103.4	-119.8	2,040	622	2,883

Difference (post-CR)	-63.8	-19.0	-1,221	74	-1,081

Abbreviations: CR, cardiac rehabilitation; DID, difference-in-difference

[*]Difference-in-differences (DID) analyses were used to compare differences in inpatient hospitalizations, ED visits, and expenditures before (2014–2015) and after the CR period (2018–2019; two-year CR period=2016–2017) between CR participants and non-participants (reference group).

[†]Total medical expenditures includes Medicare and out-of-pocket expenditures

[‡]Controlled for age, sex, race and ethnicity, dual enrolment status, urbanicity, U.S. Census Division, comorbidities, CR-qualifying event (AMI [with or without procedure], CABG [with or without AMI], combination event [with or without AMI], Heart valve repair or replacement [with or without AMI], PCI [with or without AMI]), HCC risk score, and primary qualifying event LOS

Table S4. Association between CR participation and inpatient hospitalizations and emergency department visits (per 1,000 persons) and expenditures (per person), for PCI (with or without AMI or other procedures) (adjusted DID estimates)[*]

	**Inpatient Hospitalizations**	**Emergency Department Visits**	**Medicare Expenditures, $**	**Out-of-pocket Expenditures, $**	**Total Medical Expenditures[†], $**
Matched cohort[‡]					
DID estimate	-57.8***	5.1	-1,268***	-10	-1,240***
Confidence interval	(-71.9 - -43.7)	(-15.2 - 25.3)	(-1,697 - -840)	(-85 - 66)	(-1,726 - -754)
p-value	<0.001	0.624	<0.001	0.799	<0.001
Beneficiaries	37,200	37,200	37,200	37,200	37,200
R-squared	0.123	0.085	0.164	0.145	0.176
Mean control (pre-CR)	-286.1	-270.0	-4761	-502	-5285
Mean treated (pre-CR)	-280.5	-277.5	-4339	-374	-4696
Difference (pre-CR)	5.6	-7.5	422	128	590
Mean control (post-CR)	-34.5	-19.9	4391	669	5236
Mean treated (post-CR)	-86.7	-22.3	3545	787	4586
Difference (post-CR)	-52.2	-2.4	-846	119	-650
Unmatched cohort[‡]					
DID estimate	-77.5***	-4.3	-1,808***	-7	-1,776***
Confidence interval	(-89.4 - -65.6)	(-22.1 - 13.4)	(-2,155 - -1,462)	(-65 - 51)	(-2,167 - -1,386)
p-value	<0.001	0.633	<0.001	0.801	<0.001
Beneficiaries	65,682	65,682	65,682	65,682	65,682
R-squared	0.129	0.09	0.184	0.156	0.193
Mean control (pre-CR)	-453.7	-312.2	-13,326	-1,716	-15,175
Mean treated (pre-CR)	-438.1	-317.2	-12,544	-1,600	-14,250
Difference (pre-CR)	15.5	-4.9	782	116	925
Mean control (post-CR)	-184.9	-47.3	-3,745	-548	-4,239
Mean treated (post-CR)	-246.9	-56.6	-4,771	-440	-5,090
Difference (post-CR)	-62.0	-9.2	-1,026	109	-851

Abbreviations: AMI, acute myocardial infarction; CR, cardiac rehabilitation; DID, difference-in-difference; PCI, percutaneous coronary intervention
[*]Difference-in-differences (DID) analyses were used to compare differences in inpatient hospitalizations, ED visits, and expenditures before (2014–2015) and after the CR period (2018–2019; two-year CR period=2016–2017) between CR participants and non-participants (reference group).<br>[†]Total medical expenditures includes Medicare and out-of-pocket expenditures<br>[‡]Controlled for age, sex, race and ethnicity, dual enrolment status, urbanicity, U.S. Census Division, comorbidities, HCC risk score, and primary qualifying event LOS

Table S5. Association between CR participation and inpatient hospitalizations and emergency department visits (per 1,000 persons) and expenditures (per person), for AMI (with or without procedure) (adjusted DID estimates)*

	**Inpatient Hospitalizations**	**Emergency Department Visits**	**Medicare Expenditures, $**	**Out-of-pocket Expenditures, $**	**Total Medical Expenditures†, $**
Matched cohort‡					
DID estimate	-69.5***	11.6	-1,862***	-70	-1,870***
Confidence interval	(-90.0 - -50.0)	(-16.5 - 39.6)	(-2,484 - -1,239)	(-169 - 30)	(-2,572 - -1,167)
p-value	<0.001	0.418	<0.001	0.169	<0.001
Beneficiaries	19,316	19,316	19,316	19,316	19,316
R-squared	0.132	0.090	0.166	0.172	0.180
Mean control (pre-CR)	-310.6	-271.7	-3,563	-347	-3,908
Mean treated (pre-CR)	-310.4	-294.7	-3,024	-196	-3,182
Difference (pre-CR)	0.2	-23.0	539	151	726
Mean control (post-CR)	-28.9	11.7	6,469	918	7,590
Mean treated (post-CR)	-98.1	0.3	5,146	999	6,446
Difference (post-CR)	-69.2	-11.4	-1,323	81	-1,144
Unmatched cohort‡					
DID estimate	-114.8***	-3.4	-3,398***	-189***	-3,524***
Confidence interval	(-131.0 - -98.7)	(-29.9 - 23.1)	(-3,858 - -2,938)	(-262 - -117)	(-4,042 - -3,007)
p-value	<0.001	0.802	<0.001	<0.001	<0.001
Beneficiaries	46,021	46,021	46,021	46,021	46,021
R-squared	0.143	0.080	0.191	0.170	0.199
Mean control (pre-CR)	-348.0	-359.9	-8,093	-731	-8,802
Mean treated (pre-CR)	-330.9	-368.9	-6,959	-573	-7,501
Difference (pre-CR)	17.1	-9.1	1,133	158	1,301
Mean control (post-CR)	-34.2	-66.9	3,045	628	3,876
Mean treated (post-CR)	-131.9	-79.3	781	597	1,653
Difference (post-CR)	-97.7	-12.5	-2,264	-31	-2,223

Abbreviations: AMI, acute myocardial infarction; CR, cardiac rehabilitation; DID, difference-in-difference
[*]Difference-in-differences (DID) analyses were used to compare differences in inpatient hospitalizations, ED visits, and expenditures before (2014–2015) and after the CR period (2018–2019; two-year CR period=2016–2017) between CR participants and non-participants (reference group).<br>[†]Total medical expenditures includes Medicare and out-of-pocket expenditures<br>[‡]Controlled for age, sex, race and ethnicity, dual enrolment status, urbanicity, U.S. Census Division, comorbidities, HCC risk score, and primary qualifying event LOS

**Table S6.** Association between CR participation and inpatient hospitalizations and emergency department visits (per 1,000 persons) and expenditures (per person), for CABG (with or without AMI or other procedures) (adjusted DID estimates)*					
	**Inpatient Hospitalizations**	**Emergency Department Visits**	**Medicare Expenditures, $**	**Out-of-pocket Expenditures, $**	**Total Medical Expenditures†, $**
Matched cohort‡					
DID estimate	-40.8***	7.5	-1,128**	-150*	-1,259**
Confidence interval	(-65.0 - -16.5)	(-25.9 - 40.9)	(-1,916 - -341)	(-282 - -19)	(-2,157 - -361)
p-value	<0.001	0.659	0.005	0.025	0.006
Beneficiaries	10,108	10,108	10,108	10,108	10,108
R-squared	0.107	0.073	0.146	0.145	0.156
Mean control (pre-CR)	-313.7	-231.3	-7,268	-1,085	-8,358
Mean treated (pre-CR)	-305.0	-234.1	-6,737	-931	-7,640
Difference (pre-CR)	8.8	-2.8	530	154	718
Mean control (post-CR)	-114.0	-30.8	741	17	930
Mean treated (post-CR)	-146.0	-26.0	143	21	389
Difference (post-CR)	-32.0	4.8	-598	4	-541
Unmatched cohort‡					
DID estimate	-52.2***	-13.2	-1,993***	-193***	-2,155***
Confidence interval	(-70.1 - -34.3)	(-40.8 - 14.3)	(-2,549 - -1,436)	(-284 - -102)	(-2,787 - -1,522)
p-value	<0.001	0.347	<0.001	<0.001	<0.001
Beneficiaries	20,158	20,158	20,158	20,158	20,158
R-squared	0.104	0.074	0.152	0.150	0.161
Mean control (pre-CR)	-285.4	-227.2	-8,429	-1,181	-9,676
Mean treated (pre-CR)	-275.0	-229.4	-7,428	-954	-8,408
Difference (pre-CR)	10.4	-2.2	1,001	227	1,269
Mean control (post-CR)	-77.5	0.4	40	-43	81
Mean treated (post-CR)	-119.4	-15.0	-953	-9	-805
Difference (post-CR)	-41.9	-15.4	-992	34	-886

Abbreviations: AMI, acute myocardial infarction; CABG, coronary artery bypass graft; CR, cardiac rehabilitation; DID, difference-in-difference
[*]Difference-in-differences (DID) analyses were used to compare differences in inpatient hospitalizations, ED visits, and expenditures before (2014–2015) and after the CR period (2018–2019; two-year CR period=2016–2017) between CR participants and non-participants (reference group).<br>[†]Total medical expenditures includes Medicare and out-of-pocket expenditures<br>[‡]Controlled for age, sex, race and ethnicity, dual enrolment status, urbanicity, U.S. Census Division, comorbidities, HCC risk score, and primary qualifying event LOS

**Table S7.** Association between CR participation and inpatient hospitalizations and emergency department visits (per 1,000 persons) and expenditures (per person), for heart valve repair or replacement (with or without AMI or other procedures) (adjusted DID estimates)*					
	**Inpatient Hospitalizations**	**Emergency Department Visits**	**Medicare Expenditures, $**	**Out-of-pocket Expenditures, $**	**Total Medical Expenditures†, $**
Matched cohort‡					
DID estimate	-17.1	-24.7	115	42	203
Confidence interval	(-44.7 - 10.5)	(-60.1 - 10.7)	(-725 - 955)	(-89 - 173)	(-746 - 1,152)
p-value	0.225	0.171	0.788	0.526	0.675
Beneficiaries	9,708	9,708	9,708	9,708	9,708
R-squared	0.112	0.070	0.145	0.156	0.157
Mean control (pre-CR)	-328.2	-283.0	-8,645	-969	-9,649
Mean treated (pre-CR)	-328.5	-266.1	-8,395	-833	-9,247
Difference (pre-CR)	-0.4	16.9	249	136	402
Mean control (post-CR)	-146.9	-74.1	-1,796	-129	-1,813
Mean treated (post-CR)	-164.3	-81.9	-1,432	49	-1,209
Difference (post-CR)	-17.5	-7.8	364	179	604
Unmatched cohort‡					
DID estimate	-35.8***	-13.0	-1,477***	-57	-1,526***
Confidence interval	(-57 - -15)	(-41 - 15)	(-2,103 - -852)	(-154 - 39)	(-2,229 - -823)
p-value	0.001	0.364	<0.001	0.243	<0.001
Beneficiaries	18,852	18,852	18,852	18,852	18,852
R-squared	0.113	0.077	0.152	0.147	0.160
Mean control (pre-CR)	-323.5	-285.4	-7,811	-609	-8,451
Mean treated (pre-CR)	-321.8	-281.9	-7,304	-484	-7,801
Difference (pre-CR)	1.8	3.5	507	125	650
Mean control (post-CR)	-127.6	-71.1	57	264	440
Mean treated (post-CR)	-161.6	-80.5	-914	331	-435

Difference (post-CR)	-34.1	-9.5	-971	67	-876

Abbreviations: AMI, acute myocardial infarction; CR, cardiac rehabilitation; DID, difference-in-difference

*Difference-in-differences (DID) analyses were used to compare differences in inpatient hospitalizations, ED visits, and expenditures before (2014–2015) and after the CR period (2018–2019; two-year CR period=2016–2017) between CR participants and non-participants (reference group).

†Total medical expenditures includes Medicare and out-of-pocket expenditures

‡Controlled for age, sex, race and ethnicity, dual enrolment status, urbanicity, U.S. Census Division, comorbidities, HCC risk score, and primary qualifying event LOS

Table S8. Association between CR participation and inpatient hospitalizations and emergency department visits (per 1,000 persons) and expenditures (per person), for combination event (with or without AMI) (adjusted DID estimates)*

	**Inpatient Hospitalizations**	**Emergency Department Visits**	**Medicare Expenditures, $**	**Out-of-pocket Expenditures, $**	**Total Medical Expenditures†, $**
Matched cohort‡					
DID estimate	-70.7***	7.2	-1,901***	-93	-1,951***
Confidence interval	(-90.5 - -50.9)	(-20.6 - 35.0)	(-2,516 - -1,287)	(-192 - 7)	(-2,645 - -1,256)
p-value	<0.001	0.612	<0.001	0.067	<0.001
Beneficiaries	18,428	18,428	18,428	18,428	18,428
R-squared	0.126	0.088	0.159	0.165	0.172
Mean control (pre-CR)	-308.7	-242.9	-3,214	-327	-3,536
Mean treated (pre-CR)	-308.1	-266.4	-2,680	-196	-2,839
Difference (pre-CR)	0.7	-23.5	535	131	697
Mean control (post-CR)	-48.0	28.6	6,146	868	7,219
Mean treated (post-CR)	-118.0	12.3	4,780	906	5,965
Difference (post-CR)	-70.0	-16.3	-1,367	38	-1,254
Unmatched cohort‡					
DID estimate	-105.9***	-15.5	-3,012***	-175***	-3,141***
Confidence interval	(-121.6 - -90.3)	(-39.8 - 8.7)	(-3,471 - -2,554)	(-247 - -102)	(-3,658 - -2,624)
p-value	<0.001	0.209	<0.001	<0.001	<0.001
Beneficiaries	32,287	32,287	32,287	32,287	32,287
R-squared	0.130	0.089	0.175	0.170	0.185
Mean control (pre-CR)	-557.8	-767.7	-16,039	-2,665	-19,027
Mean treated (pre-CR)	-548.3	-783.1	-15,141	-2,505	-17,948
Difference (pre-CR)	9.5	-15.4	898	160	1,079
Mean control (post-CR)	-273.7	-478.6	-6,029	-1,430	-7,614
Mean treated (post-CR)	-370.2	-509.6	-8,143	-1,445	-9,676
Difference (post-CR)	-96.5	-30.9	-2,114	-15	-2,062

Abbreviations: AMI, acute myocardial infarction; CR, cardiac rehabilitation; DID, difference-in-difference
[*]Difference-in-differences (DID) analyses were used to compare differences in inpatient hospitalizations, ED visits, and expenditures before (2014–2015) and after the CR period (2018–2019; two-year CR period=2016–2017) between CR participants and non-participants (reference group).<br>[†]Total medical expenditures includes Medicare and out-of-pocket expenditures<br>[‡]Controlled for age, sex, race and ethnicity, dual enrolment status, urbanicity, U.S. Census Division, comorbidities, HCC risk score, and primary qualifying event LOS

**Table S9.** Association between amount of CR participation and subsequent annual inpatient hospitalizations (per 1,000 persons), for any CR-qualifying event (adjusted DID estimates)*				
	**Low (2-11 sessions)**	**Medium (12-23 sessions)**	**High (24-35 sessions)**	**Completion (≥36 sessions)**
Matched cohort				
DID estimate	-9.0	-43.3***	-54.6***	-60.8***
Confidence interval	(-32.4 - 14.4)	(-63.3 - -23.4)	(-70.7 - -38.5)	(-77.4 - -44.2)
p-value	0.450	<0.001	<0.001	<0.001
Beneficiaries	4,209	5,868	9,837	8,920
R-squared	0.126	0.126	0.122	0.123
Mean control (pre-CR)	-350.8	-334.4	-323.7	-317.0
Mean treated (pre-CR)	-360.2	-322.6	-320.2	-308.2
Difference (pre-CR)	-9.4	11.7	3.6	8.8
Mean control (post-CR)	-113.2	-96.8	-86.2	-79.5
Mean treated (post-CR)	-131.7	-128.4	-137.2	-131.5
Difference (post-CR)	-18.4	-31.6	-51.0	-52.0
Unmatched cohort				
DID estimate	-45.2***	-80.9***	-93.6***	-105.3***
Confidence interval	(-66.4 - -23.9)	(-98.6 - -63.1)	(-107.6 - -79.7)	(-119.9 - -90.7)
p-value	<0.001	<0.001	<0.001	<0.001
Beneficiaries	5,819	8,397	14,163	12,686
R-squared	0.133	0.134	0.133	0.133
Mean control (pre-CR)	-326.1	-314.5	-319.5	-301.2
Mean treated (pre-CR)	-313.4	-279.3	-294.6	-272.4
Difference (pre-CR)	12.7	35.2	25.0	28.8
Mean control (post-CR)	-57.2	-45.7	-50.7	-32.3
Mean treated (post-CR)	-89.7	-91.4	-119.4	-108.8
Difference (post-CR)	-32.5	-45.7	-68.7	-76.5

Abbreviations: CR, cardiac rehabilitation; DID, difference-in-difference
*Difference-in-differences (DID) analyses were used to compare differences in inpatient hospitalizations before (2014–2015) and after the CR period (2018–2019); two-year CR period=2016–2017) between CR participants (by amount of CR participation: 2–11, 12–23, 24–35, ≥36 CR sessions) and non-participants (reference group [n=28,834, 50.0% for the matched cohort and n=76,146, 65.0% for the unmatched cohort]). All models controlled for age, sex, race and ethnicity, dual enrolment status, urbanicity, U.S. Census Division, comorbidities, CR-qualifying event (AMI [with or without procedure], CABG [with or without AMI], combination event [with or without AMI], Heart valve repair or replacement [with or without AMI], PCI [with or without AMI]), HCC risk score, and primary qualifying event LOS.

Table S10. Association between amount of CR participation and subsequent annual emergency department visits (per 1,000 persons), for any CR-qualifying event (adjusted DID estimates)[*]

	**Low (2-11 sessions)**	**Medium (12-23 sessions)**	**High (24-35 sessions)**	**Completion (≥36 sessions)**
Matched cohort				
DID estimate	31.9	-5.4	3.1	-8.7
Confidence interval	(-0.9 - 64.6)	(-33.2 - 22.4)	(-19.3 - 25.4)	(-31.8 - 14.4)
p-value	0.056	0.705	0.788	0.461
Beneficiaries	4,209	5,868	9,837	8,920
R-squared	0.084	0.083	0.081	0.080
Mean control (pre-CR)	-377.9	-333.4	-318.8	-362.2
Mean treated (pre-CR)	-342.9	-318.6	-326.2	-390.3
Difference (pre-CR)	35.1	14.8	-7.4	-28.1
Mean control (post-CR)	-139.3	-94.8	-80.2	-123.6
Mean treated (post-CR)	-72.4	-85.3	-84.6	-160.3
Difference (post-CR)				
Unmatched cohort				
DID estimate	7.2	-29.4*	-19.2	-34.1**
Confidence interval	(-25.9 - 40.3)	(-57.1 - -1.8)	(-40.8 - 2.5)	(-56.7 - -11.4)
p-value	0.671	0.037	0.082	0.003
Beneficiaries	5,819	8,397	14,163	12,686
R-squared	0.082	0.082	0.082	0.082
Mean control (pre-CR)	-383.6	-372.9	-347.8	-362.5
Mean treated (pre-CR)	-345.5	-355.6	-350.4	-379.2
Difference (pre-CR)	38.1	17.3	-2.6	-16.7
Mean control (post-CR)	-122.9	-112.2	-87.1	-101.8
Mean treated (post-CR)	-77.7	-124.4	-108.9	-152.6
Difference (post-CR)	45.3	-12.2	-21.8	-50.8

Abbreviations: CR, cardiac rehabilitation; DID, difference-in-difference
*Difference-in-differences (DID) analyses were used to compare differences in emergency department visits before (2014–2015) and after the CR period (2018–2019); two-year CR period=2016–2017) between CR participants (by amount of CR participation: 2–11, 12–23, 24–35, ≥36 CR sessions) and non-participants (reference group [n=28,834, 50.0% for the matched cohort and n=76,146, 65.0% for the unmatched cohort]). All models controlled for age, sex, race and ethnicity, dual enrolment status, urbanicity, U.S. Census Division, comorbidities, CR-qualifying event (AMI [with or without procedure], CABG [with or without AMI], combination event [with or without AMI], Heart valve repair or replacement [with or without AMI], PCI [with or without AMI]), HCC risk score, and primary qualifying event LOS.

**Table S11.** Association between amount of CR participation and subsequent annual Medicare expenditures (per person), for any CR-qualifying event (adjusted DID estimates)*				
	**Low (2-11 sessions)**	**Medium (12-23 sessions)**	**High (24-35 sessions)**	**Completion (≥36 sessions)**
Matched cohort				
DID estimate	55	-1,201***	-1,033***	-1,347***
Confidence interval	(-664 - 774)	(-1,814 - -588)	(-1,535 - -532)	(-1,861 - -833)
p-value	0.880	<0.001	<0.001	<0.001
Beneficiaries	4,209	5,868	9,837	8,920
R-squared	0.160	0.160	0.155	0.158
Mean control (pre-CR)	-6,815	-6,426	-5,477	-7,386
Mean treated (pre-CR)	-6,746	-5,928	-5,022	-6,923
Difference (pre-CR)	69	498	455	463
Mean control (post-CR)	1,973	2,363	3,311	1,403
Mean treated (post-CR)	2,097	1,660	2,733	518
Difference (post-CR)	124	-703	-578	-885
Unmatched cohort				
DID estimate	-1,324***	-2,531***	-2,562***	-2,815***
Confidence interval	(-1,935 - -712)	(-3,044 - -2,019)	(-2,968 - -2,156)	(-3,239 - -2,392)
p-value	<0.001	<0.001	<0.001	<0.001
Beneficiaries	5,819	8,397	14,163	12,686
R-squared	0.184	0.184	0.181	0.183
Mean control (pre-CR)	-7,382	-7,230	-6,047	-6,837
Mean treated (pre-CR)	-6,500	-5,763	-4,739	-5,520
Difference (pre-CR)	882	1,467	1,308	1,317
Mean control (post-CR)	2,427	2,579	3,762	2,972
Mean treated (post-CR)	1,985	1,515	2,508	1,473
Difference (post-CR)	-442	-1,065	-1,254	-1,499

Abbreviations: CR, cardiac rehabilitation; DID, difference-in-difference
[*]Difference-in-differences (DID) analyses were used to compare differences in Medicare expenditures before (2014–2015) and after the CR period (2018–2019); two-year CR period=2016–2017) between CR participants (by amount of CR participation: 2–11, 12–23, 24–35, ≥36 CR sessions) and non-participants (reference group [n=28,834, 50.0% for the matched cohort and n=76,146, 65.0% for the unmatched cohort]). All models controlled for age, sex, race and ethnicity, dual enrolment status, urbanicity, U.S. Census Division, comorbidities, CR-qualifying event (AMI [with or without procedure], CABG [with or without AMI], combination event [with or without AMI], Heart valve repair or replacement [with or without AMI], PCI [with or without AMI]), HCC risk score, and primary qualifying event LOS.

Table S12. Association between amount of CR participation and subsequent annual out-of-pocket expenditures (per person), for any CR-qualifying event (adjusted DID estimates)[*]

	**Low (2-11 sessions)**	**Medium (12-23 sessions)**	**High (24-35 sessions)**	**Completion (≥36 sessions)**
Matched cohort				
DID estimate	94	-48	-27	-43
Confidence interval	(-31 - 219)	(-147 - 51)	(-108 - 54)	(-126 - 39)
p-value	0.141	0.345	0.519	0.305
Beneficiaries	4,209	5,868	9,837	8,920
R-squared	0.135	0.154	0.153	0.155
Mean control (pre-CR)	-1,134	-924	-768	-778
Mean treated (pre-CR)	-1,009	-767	-618	-655
Difference (pre-CR)	125	157	150	123
Mean control (post-CR)	-9	201	357	347
Mean treated (post-CR)	210	310	481	427
Difference (post-CR)	219	109	124	79
Unmatched cohort				
DID estimate	-45	-155***	-135***	-158***
Confidence interval	(-144 - 55)	(-236 - -74)	(-198 - -71)	(-224 - -91)
p-value	0.377	<0.001	<0.001	<0.001
Beneficiaries	5,819	8,397	14,163	12,686
R-squared	0.150	0.158	0.159	0.160
Mean control (pre-CR)	-811	-673	-563	-551
Mean treated (pre-CR)	-599	-439	-365	-376
Difference (pre-CR)	212	234	198	175
Mean control (post-CR)	381	519	629	641
Mean treated (post-CR)	548	598	692	658
Difference (post-CR)	167	79	64	17

Abbreviations: CR, cardiac rehabilitation; DID, difference-in-difference
*Difference-in-differences (DID) analyses were used to compare differences in out-of-pocket expenditures before (2014–2015) and after the CR period (2018–2019); two-year CR period=2016–2017) between CR participants (by amount of CR participation: 2–11, 12–23, 24–35, ≥36 CR sessions) and non-participants (reference group [n=28,834, 50.0% for the matched cohort and n=76,146, 65.0% for the unmatched cohort]). All models controlled for age, sex, race and ethnicity, dual enrolment status, urbanicity, U.S. Census Division, comorbidities, CR-qualifying event (AMI [with or without procedure], CABG [with or without AMI], combination event [with or without AMI], Heart valve repair or replacement [with or without AMI], PCI [with or without AMI]), HCC risk score, and primary qualifying event LOS.

Table S13. Association between amount of CR participation and subsequent annual total medical expenditures (per person), for any CR-qualifying event (adjusted DID estimates)*

	**Low (2-11 sessions)**	**Medium (12-23 sessions)**	**High (24-35 sessions)**	**Completion (≥36 sessions)**
Matched cohort				
DID estimate	133	-1,228***	-996***	-1,329***
Confidence interval	(-682 - 947)	(-1,922 - -535)	(-1,563 - -430)	(-1,911 - -748)
p-value	0.750	<0.001	<0.001	<0.001
Beneficiaries	4,209	5,868	9,837	8,920
R-squared	0.170	0.171	0.167	0.169
Mean control (pre-CR)	-8,043	-7,414	-6,284	-8,119
Mean treated (pre-CR)	-7,807	-6,714	-5,642	-7,507
Difference (pre-CR)	236	700	641	612
Mean control (post-CR)	2,057	2,686	3,816	1,981
Mean treated (post-CR)	2,426	2,158	3,461	1,264
Difference (post-CR)	369	-528	-355	-718
Unmatched cohort				
DID estimate	-1,384***	-2,690***	-2,647***	-2,928***
Confidence interval	(-2,072 - -696)	(-3,267 - -2,114)	(-3,104 - -2,190)	(-3,404 - -2,452)
p-value	<0.001	<0.001	<0.001	<0.001
Beneficiaries	5,819	8,397	14,163	12,686
R-squared	0.190	0.191	0.188	0.190
Mean control (pre-CR)	-8,220	-7,912	-6,630	-7,359
Mean treated (pre-CR)	-7,090	-6,166	-5,087	-5,843
Difference (pre-CR)	1,130	1,747	1,543	1,515
Mean control (post-CR)	2,961	3,269	4,552	3,823
Mean treated (post-CR)	2,708	2,326	3,448	2,410
Difference (post-CR)	-254	-944	-1,104	-1,413

Abbreviations: CR, cardiac rehabilitation; DID, difference-in-difference
*Difference-in-differences (DID) analyses were used to compare differences in total medical expenditures before (2014–2015) and after the CR period (2018–2019); two-year CR period=2016–2017) between CR participants (by amount of CR participation: 2–11, 12–23, 24–35, ≥36 CR sessions) and non-participants (reference group [n=28,834, 50.0% for the matched cohort and n=76,146, 65.0% for the unmatched cohort]). All models controlled for age, sex, race and ethnicity, dual enrolment status, urbanicity, U.S. Census Division, comorbidities, CR-qualifying event (AMI [with or without procedure], CABG [with or without AMI], combination event [with or without AMI], Heart valve repair or replacement [with or without AMI], PCI [with or without AMI]), HCC risk score, and primary qualifying event LOS.

Table S14. Association between CR participation and the top Medicare expenditures (per person), for any CR-qualifying event (adjusted DID estimates) (matched cohort)[*,†]

	Inpatient	Hospital Outpatient	Part B	Part D	Skilled Nursing Facility
DID estimate	-685***	228***	-116	82	-260***
Confidence Interval	(-881 - -489)	(93 - 363)	(-249 - 17)	(-81 - 245)	(-327 - -194)
p-value	<0.001	0.001	0.087	0.324	<0.001
Beneficiaries	57,668	57,668	57,668	57,668	57,668
R-squared	0.076	0.056	0.089	0.049	0.045
Mean control (pre-CR)	-3,159	71	-1,352	-648	-1,337
Mean treated (pre-CR)	-3,053	236	-1,145	-593	-1,330
Difference (pre-CR)	106	165	207	55	7
Mean control (post-CR)	393	1,727	437	806	-477
Mean treated (post-CR)	-187	2,120	529	943	-731
Difference (post-CR)	-580	393	91	137	-253

Abbreviations: CR, cardiac rehabilitation; DID, difference-in-difference

[*]Difference-in-differences (DID) analyses were used to compare differences in inpatient hospitalizations, ED visits, and expenditures before (2014–2015) and after the CR period (2018–2019; two-year CR period=2016–2017) between CR participants and non-participants (reference group).

[†] Controlled for age, sex, race and ethnicity, dual enrolment status, urbanicity, U.S. Census Division, comorbidities, CR-qualifying event (AMI [with or without procedure], CABG [with or without AMI], combination event [with or without AMI], Heart valve repair or replacement [with or without AMI], PCI [with or without AMI]), HCC risk score, and primary qualifying event LOS.

Table S15. Association between CR participation and cardiovascular disease-related inpatient hospitalizations and emergency department visits (per 1,000 persons) and expenditures (per person), for any CR-qualifying event (adjusted DID estimates)[*]

	**Inpatient Hospitalizations**	**Emergency Department Visits**	**Total Medical Expenditures, $**
Matched cohort			
DID estimate	-14.6***	0.1	-273***
Confidence Interval	(-20.4 - -9.0)	(-3.8 - 3.9)	(-427 - -119)
p-value	<0.001	0.950	<0.001
Beneficiaries	57,668	57,668	57,668
R-squared	0.055	0.019	0.066
Mean control (pre-CR)	-111.2	-52.8	-2,626
Mean treated (pre-CR)	-111.1	-52.7	-2,708
Difference (pre-CR)	0.2	0.1	-81.4
Mean control (post-CR)	-15.4	-24.3	485
Mean treated (post-CR)	-29.9	-24.1	130
Difference (post-CR)	-14.5	0.2	-354
Unmatched cohort			
DID estimate	-36.3***	-3.6*	-918***
Confidence Interval	(-40.9 - -31.8)	(-6.7 - -0.6)	(-1,036 - -799)
p-value	<0.001	0.019	<0.001
Beneficiaries	117,211	117,211	117,211
R-squared	0.061	0.021	0.071
Mean control (pre-CR)	-126.8	-69.7	-3,025
Mean treated (pre-CR)	-116.4	-67.5	-2,777
Difference (pre-CR)	10.4	2.2	248
Mean control (post-CR)	-16.0	-37.0	472
Mean treated (post-CR)	-42.0	-38.5	-197
Difference (post-CR)	-25.9	-1.5	-670

Abbreviations: CR, cardiac rehabilitation; DID, difference-in-difference

[*]Difference-in-differences (DID) analyses were used to compare differences in inpatient hospitalizations, emergency department visits, and expenditures before (2014–2015) and after the CR period (2018–2019; two-year CR period=2016–2017) between CR participants and non-participants (reference group). We controlled for age, sex, race and ethnicity, dual enrolment status, urbanicity, U.S. Census Division, comorbidities, CR-qualifying event (AMI [with or without procedure], CABG [with or without AMI], combination event [with or without AMI], Heart valve repair or replacement [with or without AMI], PCI [with or without AMI]), HCC risk score, and primary qualifying event LOS in all models.

Table S16. Association between CR participation and inpatient hospitalizations and emergency department visits (per 1,000 persons) and expenditures (per person), for any CR-qualifying event for women vs. men (adjusted triple difference estimates for the matched cohort)*

	Women vs. Men[b,c] Ratio (95% CI)	p-value
**Inpatient Hospitalizations**	1.0033 (0.9710, 1.0366)	0.8844
**Emergency Department Visits**	1.0076 (0.9780, 1.0381)	0.6194
**Medicare Expenditures, $**	0.9706 (0.9343, 1.0080)	0.1246
**Out-of-pocket Expenditures, $**	0.9841 (0.9563, 1.0127)	0.2724
**Total Medical Expenditures[d], $**	0.9772 (0.9388, 1.0068)	0.1142

Abbreviations: CI, confidence interval; CR, cardiac rehabilitation

*Triple difference models were used to compare differences in inpatient hospitalizations, ED visits, and expenditures before (2014–2015) and after the CR period (2018–2019; two-year CR period=2016–2017) between CR participants and non-participants (reference group) and women and men (reference group).

†Controlled for age, race and ethnicity, dual enrolment status, urbanicity, U.S. Census Division, comorbidities, CR-qualifying event (AMI [with or without procedure], CABG [with or without AMI], combination event [with or without AMI], Heart valve repair or replacement [with or without AMI], PCI [with or without AMI]), HCC risk score, and primary qualifying event LOS

‡To obtain the triple difference estimates, we used generalized linear models in SAS.

§Total medical expenditures includes Medicare and out-of-pocket expenditures

Table S17. Association between CR participation and inpatient hospitalizations and emergency department visits (per 1,000 persons) and expenditures (per person), for any CR-qualifying event including year 2020 (adjusted DID estimates)[*]

	**Inpatient Hospitalizations**	**Emergency Department Visits**	**Medicare Expenditures, $**	**Out-of-pocket Expenditures, $**	**Total Medical Expenditures[†], $**
Matched cohort[‡]					
DID estimate	-43.3***	6.5	-874.3***	-28	-906***
Confidence interval	(-54.2 - -32.4)	(-8.6 - 21.6)	(-1,234 - -515)	(-82 - 26)	(-1,306 - -506)
p-value	<0.001	0.398	<0.001	0.309	<0.001
Beneficiaries	50,358	50,358	50,358	50,358	50,358
R-squared	0.099	0.071	0.129	0.144	0.138
Mean control (pre-CR)	-253.5	-285.1	-5,788	-579	-6,376
Mean treated (pre-CR)	-248.2	-296.2	-5,364	-430	-5,812
Difference (pre-CR)	5.3	-11.1	424.5	148	564
Mean control (post-CR)	-50.9	-100.9	2,187	405	2,588
Mean treated (post-CR)	-88.8	-105.5	1,737	525	2,246
Difference (post-CR)	-37.9	-4.6	-450	120	-342
Unmatched cohort[‡]					
DID estimate	-64.86***	-5.4	-1,954***	-80***	-2,052***
Confidence interval	(-73.4 - -56.3)	(-18.5 - 7.8)	(-2,221 - -1,687)	(-121 - -39)	(-2,350 - -1,754)
p-value	<0.001	0.425	<0.001	<0.001	<0.001
Beneficiaries	100,841	100,841	100,841	100,841	100,841
R-squared	0.104	0.069	0.149	0.139	0.154
Mean control (pre-CR)	-255.9	-302.5	-5,934	-475	-6,218
Mean treated (pre-CR)	-237.4	-306.9	-4,787	-297	-4,902
Difference (pre-CR)	18.5	-4.4	1,147	178	1,315
Mean control (post-CR)	-39.1	-106.4	2,783	526	3,508
Mean treated (post-CR)	-85.5	-116.2	1,977	625	2,771
Difference (post-CR)	-46.4	-10.0	-807	98	-737

Abbreviations: CR, cardiac rehabilitation; DID, difference-in-difference
[*]Difference-in-differences (DID) analyses were used to compare differences in inpatient hospitalizations, ED visits, and expenditures before (2014–2015) and after the CR period (2018–2020; two-year CR period=2016–2017) between CR participants and non-participants (reference group). This sensitivity analysis including year 2020 extended into the start of the COVID-19 public health emergency, which had a universal depressive effect on all forms of healthcare utilization. While the pandemic may have affected observed outcomes, it affected both CR participants and non-participants equally. From 2019 to 2020, mean inpatient hospitalizations decreased marginally for both CR participants (392 [SD, 882] to 382 [SD, 895]) and non-participants (341 [SD, 795] to 330 [SD, 798]); and mean ED visits decreased for both CR participants (574 [1,170] to 486 [1,063]) and non-participants (565 [1,126] to 466 [953]) (based on unadjusted findings).<br>[†]Total medical expenditures includes Medicare and out-of-pocket expenditures<br>[‡]Controlled for age, sex, race and ethnicity, dual enrolment status, urbanicity, U.S. Census Division, comorbidities, CR-qualifying event (AMI [with or without procedure], CABG [with or without AMI], combination event [with or without AMI], Heart valve repair or replacement [with or without AMI], PCI [with or without AMI]), HCC risk score, and primary qualifying event LOS

Table S18. Comparing expenditures between our main analysis and sensitivity analysis excluding the top 1% of expenditures for any CR-qualifying event (adjusted DID estimates)[*,†]

	Medicare Expenditures, $		Out-of-pocket Expenditures, $		Total Medical Expenditures[‡], $	
	**Main Analysis**	**Sensitivity Analysis[§]**	**Main Analysis**	**Sensitivity Analysis[§]**	**Main Analysis**	**Sensitivity Analysis[§]**
DID estimate	-1,005***	-826***	-19	-2	-982***	-793***
Confidence interval	(-1,352 - -659)	(-1,149 - -504)	(-78 - 41)	(-57 – 52)	(-1,375 - -589)	(-1,159 - -427)
p-value	<0.001	<0.001	0.539	0.932	<0.001	<0.001
Beneficiaries	57,668	57,102	57,668	57,102	57,668	57,102
R-squared	0.160	0.158	0.149	0.152	0.171	0.170
Mean control (pre-CR)	-6,374	-7234	-815	-1007	-7,244	-8277
Mean treated (pre-CR)	-5,973	-6822	-676	-869	-6,667	-7685
Difference (pre-CR)	401	412	140	138	577	592
Mean control (post-CR)	2,414	1680	310	148	2,856	1987
Mean treated (post-CR)	1,809	1265	431	284	2,452	1786
Difference (post-CR)	-604	-415	121	136	-404	-202

Abbreviations: CR, cardiac rehabilitation; DID, difference-in-difference

[*]Difference-in-differences (DID) analyses were used to compare differences in inpatient hospitalizations, ED visits, and expenditures before (2014–2015) and after the CR period (2018–2019; two-year CR period=2016–2017) between CR participants and non-participants (reference group).

[†]Controlled for age, sex, race and ethnicity, dual enrolment status, urbanicity, U.S. Census Division, comorbidities, CR-qualifying event (AMI [with or without procedure], CABG [with or without AMI], combination event [with or without AMI], Heart valve repair or replacement [with or without AMI], PCI [with or without AMI]), HCC risk score, and primary qualifying event LOS.

[‡]Total medical expenditures includes Medicare and out-of-pocket expenditures

[§]Sensitivity analysis, excluding top 1% of expenditures for the matched cohort.

Figure S1. Data attrition diagram

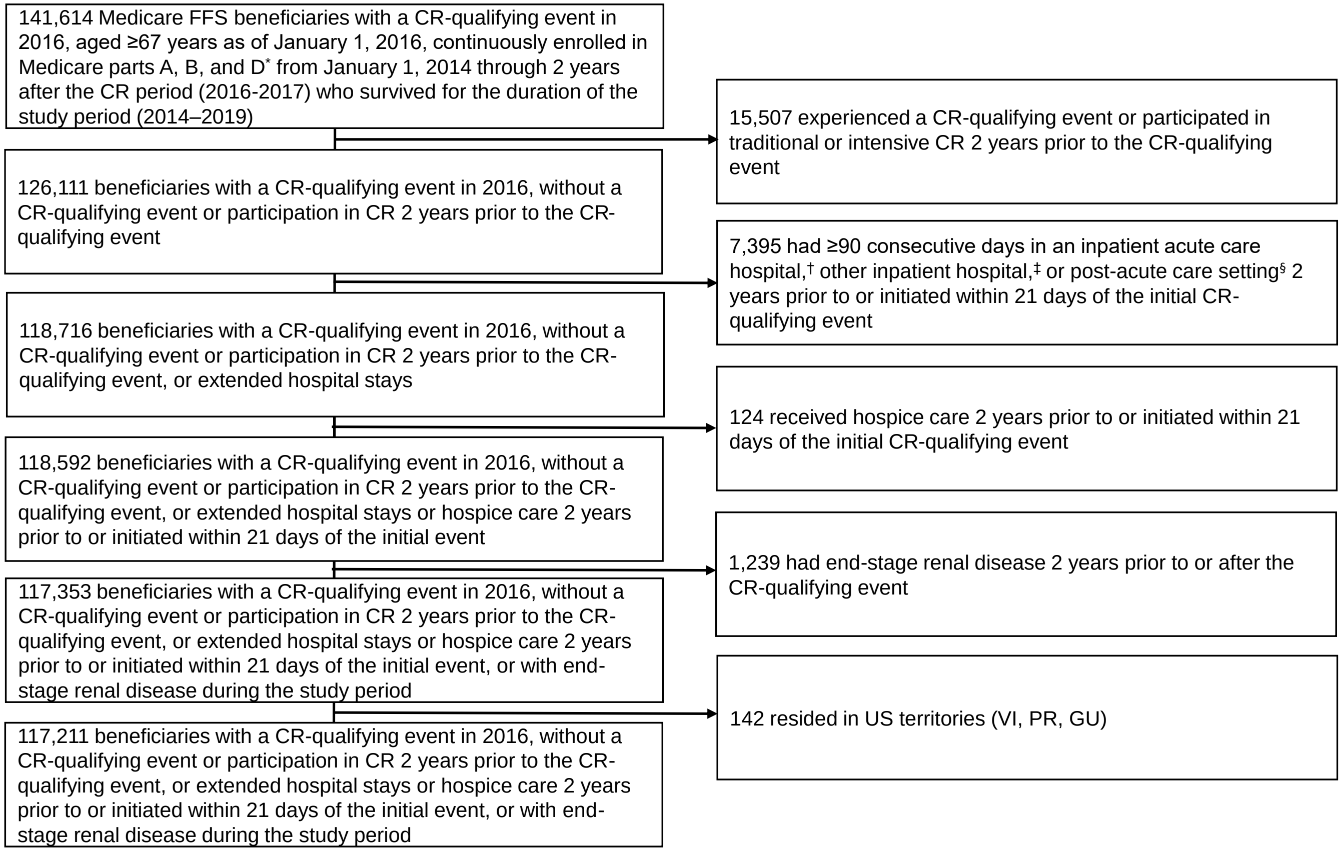

[*]Medicare Part A expenditures come from inpatient hospital stays, skilled nursing facility stays, inpatient care in a skilled nursing facility that is not custodial or long-term care, and hospice and home health care. Medicare Part B expenditures come from physicians' services, outpatient care (including CR), medical supplies (e.g., durable medical equipment), and preventative services. Medicare Part D expenditures come from prescription drug services.

[†]Inpatient acute care hospital (inpatient prospective payment system reimbursed hospital, critical access hospital)

[‡]Other inpatient hospital (inpatient psychiatric facility, other hospital type [e.g., cancer center])

[§]Post-acute care setting (long-term care hospital, inpatient rehabilitation facility, skilled nursing facility, home health)

Figure S2. Study timeline

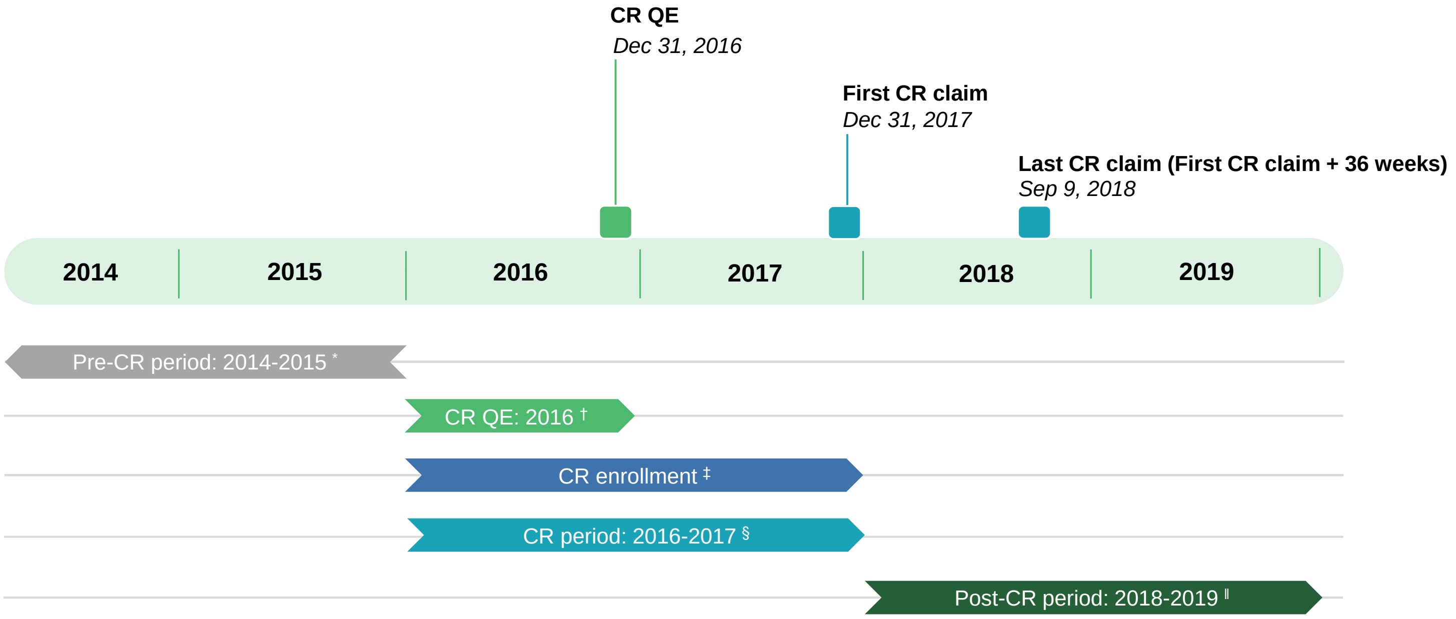

Abbreviations: CR, cardiac rehabilitation; QE, qualifying event

[*]Pre-CR period: 2-years (2014-2015)

[†]CR QEs: This study examines CR QEs that occurred in 2016

- Earliest CR QE: Jan 1, 2016
- Latest CR QE: Dec 31, 2016

[‡]CR enrollment (1st CR claim) can occur within 365 days of the CR QE index date (hospital discharge/outpatient procedure date):

- Earliest CR enrollment date: Jan 1, 2016 (i.e., if CR QE = Jan 1, 2016)
- Latest CR enrollment date: Dec 31, 2017 (i.e., if CR QE = Dec 31, 2016)

[§]CR period: Beneficiaries have 36 weeks from CR enrollment to complete 36 CR sessions (1st CR claim + 36 weeks):

- Earliest CR period range (Jan 1, 2016 + 36 weeks): Jan 1, 2016-Sep 9, 2016
- Latest CR period range (Dec 31, 2017 + 36 weeks): Dec 31, 2017-Sep 9, 2018

[‖]Post-CR period: 2 years (2018-2019)

- Earliest post-CR period range: Sep 9, 2016-Sep 9, 2018
- Latest post-CR period range: Sep 9, 2018-Sep 9, 2020 (0.06% [n=71] of individuals had less than a 2-years of follow-up data)
